# Constructing Double Heterojunctions on 1T/2H-MoS_2_@Co_3_S_4_ Electrocatalysts for Regulating Li_2_O_2_ Formation in Lithium-Oxygen Batteries

**DOI:** 10.1007/s40820-025-01895-x

**Published:** 2025-09-01

**Authors:** Yichuan Dou, Zhuang Liu, Lanling Zhao, Jian Zhang, Fanpeng Meng, Yao Liu, Zidong Zhang, Xingao Li, Zheng Shang, Lu Wang, Jun Wang

**Affiliations:** 1 Key Laboratory for Liquid-Solid Structural Evolution and Processing of Materials (Ministry of Education), Shandong University, Jinan, 250061 People’s Republic of China; 2 School of Physics, Shandong University, Jinan, 250061 People’s Republic of China; 3 Shandong Guiyuan Advanced Ceramics Co., Ltd, Zibo, 255020 People’s Republic of China; 4 Shandong Key Laboratory of Metamaterial and Electromagnetic Manipulation Technology, Jinan, 250061 People’s Republic of China

**Keywords:** Double heterojunctions, d-p hybridization, Tunable Li_2_O_2_ deposition, Electrocatalysts, Lithium-oxygen batteries

## Abstract

Co_3_S_4_ electrocatalysts with mixed valences of Co ions and excellent structural stability possess favorable oxygen evolution reaction (OER) activity, yet challenges remain in fabricating rechargeable lithium-oxygen batteries (LOBs) due to their poor OER performance, resulting from poor electrical conductivity and overly strong intermediate adsorption. In this work, fancy double heterojunctions on 1T/2H-MoS_2_@Co_3_S_4_ (1T/2H-MCS) were constructed derived from the charge donation from Co to Mo ions, thus inducing the phase transformation of MoS_2_ from 2H to 1T. The unique features of these double heterojunctions endow the 1T/2H-MCS with complementary catalysis during charging and discharging processes. It is worth noting that 1T-MoS_2_@Co_3_S_4_ could provide fast Co–S–Mo electron transport channels to promote ORR/OER kinetics, and 2H-MoS_2_@Co_3_S_4_ contributed to enabling moderate e_g_ orbital occupancy when adsorbed with oxygen-containing intermediates. On the basis, the Li_2_O_2_ nucleation route was changed to solution and surface dual pathways, improving reversible deposition and decomposition kinetics. As a result, 1T/2H-MCS cathodes exhibit an improved electrocatalytic performance compared with those of Co_3_S_4_ and MoS_2_ cathodes. This innovative heterostructure design provides a reliable strategy to construct efficient transition metal sulfide catalysts by improving electrical conductivity and modulating adsorption toward oxygenated intermediates for LOBs.

**Supplementary Information:**

The online version contains supplementary material available at 10.1007/s40820-025-01895-x.

## Highlights

- 1T/2H-MoS_2_@Co_3_S_4_ electrocatalysts were constructed by interfacial charge donation from Co to Mo atoms, resulting in formation of double heterojunctions including 1T-MoS_2_@Co_3_S_4_ and 2H-MoS_2_@Co_3_S_4_.
- Complementary effect from double heterojunctions not only triggered fast charge transport on Co–S–Mo couplings, but also enabled moderate e_g_ orbital occupancy to adsorb oxygen-containing intermediates for efficient oxygen electrocatalysis.
- Optimal adsorption energies for solution and surface dual reaction pathways were achieved, forming two kinds of discharge product morphologies during cycling to enhance performance of Li–O_2_ batteries.

**Supplementary Information:**

The online version contains supplementary material available at 10.1007/s40820-025-01895-x.

## Graphical abstract

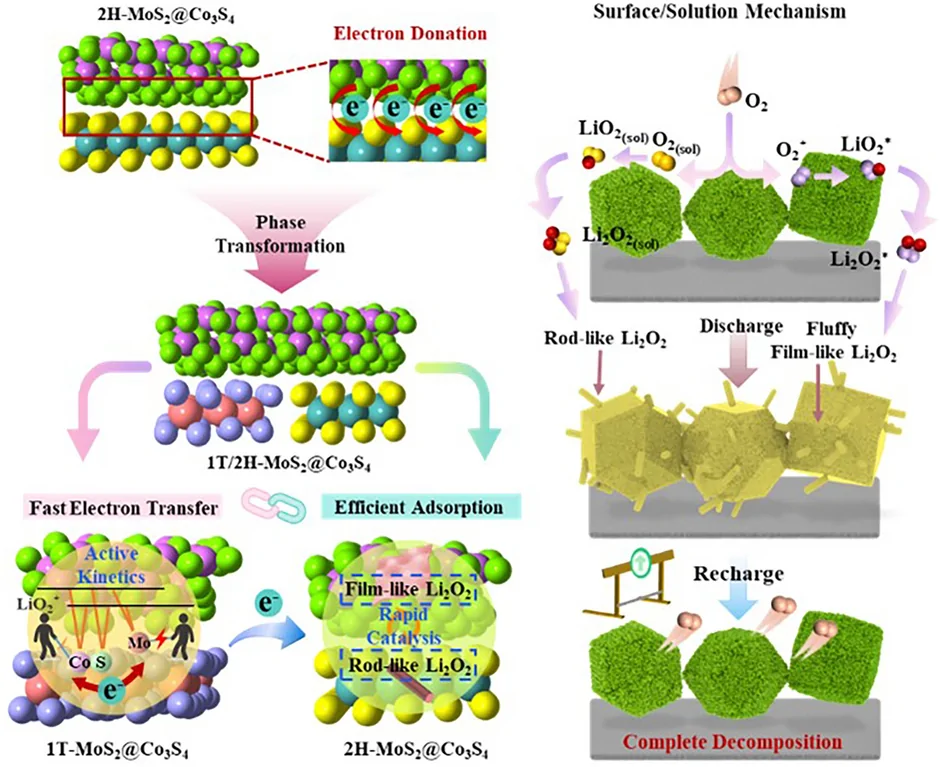

## Introduction

Aprotic lithium-oxygen batteries (LOBs) are one of the most promising candidates to replace lithium-ion batteries (LIBs) for electric vehicles, robots, and large-scale grids, because of their ultrahigh theoretical energy density (3,500 W h kg^−1^). Generally, the reaction mechanisms during cycling are based on Li^+^  + O_2_ ↔ Li_2_O_2_ (E^0^ = 2.96 V *vs.* Li/Li^+^), which involves oxygen reduction reaction (ORR) for discharging and oxygen evolution reaction (OER) for charging [1, 2]. However, such a powerful system is greatly hindered for further applications by poor rate performance, limited cyclic life, and high overpotentials. These problems mainly result from their sluggish redox kinetics, triggering the accumulation of inactive discharge product (Li_2_O_2_) and even insulated side reaction product (LiOH and Li_2_CO_3_) on the cathode [3]. Considering that the cathodes should exposure the triple-phase contact interface regions (cathode/electrolyte/O_2_) and enable discharge product storage, employing ideal cathode catalysts for fast electron transfer rate and reversible discharge product formation would greatly enhance the electrocatalytic performance and accelerate the commercial applications of LOBs.

Noble metals and their alloys exhibit high-efficiency catalytic properties in LOBs, while the high cost and scarcity on the Earth's crust seriously restrict their large-scale applications [4, 5]. Transition metal chalcogenides (TMCs) have been attracting extensive attention worldwide on account of their low cost, excellent chemically active electrocatalytic property, and diversity of structure [6]. Among them, Co_3_S_4_ possesses a typical spinel structure with a mixture valence state of Co^2+^ and Co^3+^, and this unique arrangement endows Co_3_S_4_ eye-catching recognition as the OER electrocatalyst, proved by density functional theory (DFT) calculations [7, 8]. Liu et al. [9] synthesized ultrathin Co_3_S_4_ nanosheets via a sonicated treatment, delivering high OER activities and reduced overpotentials in overall water splitting reactions. Wang et al. [10] present superior manner in OER performance, which can be realized by inducing sulfur vacancies in Co_3_S_4_. Furthermore, Yang et al. [11] demonstrate that the adsorption states of oxygen-containing intermediates could be tailored by Ni incorporation to largely influence OER activities. However, it is proposed that the monometallic sulfides generally fail to simultaneously exhibit the high electrocatalytic activities toward bifunctional electrocatalytic reactions (ORR and OER) [12]. In fact, when exposed to ambient environment or an oxygen-containing solution, Co_3_S_4_ tends to be extraordinarily reactive and unstable, which is prone to rapid oxidation due to the weak Co–S bonds and results in low electrical conductivity during ORR [13, 14]. Thus, it is necessary to construct heterostructures to offer conductive support for Co_3_S_4_ and further achieve bifunctional electrocatalytic abilities.

As another widely studied TMCs, MoS_2_ catalysts have been widely applied as one of the key alternatives to noble metal catalysts in electrochemical fields [15, 16]. It is found that MoS_2_ exhibits the layered S-Mo-S sandwich structure, connected by weak van der Waals forces along the c-axis. Besides, according to the stacking manner of S atoms, there are three different crystalline phases of MoS_2_, including 1T (trigonal structure, octahedral coordination), 2H (hexagonal structure, trigonal prismatic coordination), and 3R (rhombohedral structure, trigonal prismatic coordination) [17]. Due to intrinsic properties, 3R-MoS_2_ has been proven to be a promising material for optoelectronic and photonic devices [18, 19]. 2H-MoS_2_ is dominant and more stable in nature, showing the characteristics of a semiconductor with a large bandgap between 1.29 and 1.90 eV. Researchers have calculated and proven that 2H-MoS_2_ possesses ultra-low adsorption energy (E_ads_) for reaction intermediates and hardly adsorbs the reaction intermediates, while the stack edge planes of 2H-MoS_2_ can confine reaction species and restrict growth of the discharge product due to the ultrahigh E_ads_. To increase electrical conductivity and E_ads_ toward oxygen-containing intermediates, a variety of strategies were carried out, including hybridization with carbon materials (carbon nanotubes [20], graphene oxide [21], and other carbon materials [22, 23]), doping with heteroatoms [24, 25], and constructing S vacancies [26, 27]. The bandgap of 1T-MoS_2_ is close to zero with metallic property, exhibiting 10^7^ times higher electrical conductivity than that of 2H-MoS_2_, which is pivotal for enhancing its catalytic performance [28, 29] Theoretical calculations confirm that the octahedral coordination favors the accommodation of these extra electrons in the d orbitals of Mo atoms, gifting 1T-MoS_2_ with metallic property [30]. However, 1T-MoS_2_ is considered to be thermodynamically unfavorable and hard to realize high-yield production, leading to spontaneous phase reversion to 2H-MoS_2_ [31, 32]. It is reported that electron configuration modulation for 2H-MoS_2_ could induce 1T-MoS_2_ formation due to the electron injection to Mo 4*d*, and the existence of 2H-MoS_2_ can contribute to stabilizing 1T-MoS_2_ [33–35]. Therefore, heterostructures based on MoS_2_ are believed to offer a new platform to explore this connection owing to the abundant charge donation on the heterointerfaces.

Herein, ZIF-67 was employed as template to synthesize 1T/2H-MoS_2_@Co_3_S_4_ heterostructures (1T/2H-MCS) as bifunctional electrocatalysts for LOBs via one-pot hydrothermal method with two-step temperature-raising process. The original electron configuration of Mo 4d orbitals was changed via the electron injection by Co 3*d*, which triggered the phase transition from 2H to 1T to form double heterojunctions of 1T-MoS_2_@Co_3_S_4_ and 2H-MoS_2_@Co_3_S_4_ in 1T/2H-MCS. According to the theoretical calculations on Co_3_S_4_, 2H-MoS_2_, 1T-MoS_2_@Co_3_S_4_, and 2H-MoS_2_@Co_3_S_4_, the complementary effects on 1T/2H-MCS were intensively investigated. On the one hand, owing to the increasing density of states (DOS) at the Fermi energy level, 1T-MoS_2_@Co_3_S_4_ exhibits excellent electrical conductivity via the fast electron transport channels of Co–S–Mo bonds, while the low E_g_ orbital occupancy is unfavorable for adsorption of oxygen-containing intermediates, resulting from the large bandgap between d-band center (E_d_) of Co and Fermi level. On the other hand, due to the appropriate d-p orbital hybridization of 2H-MoS_2_@Co_3_S_4_ and LiO_2_ with modulated E_d_ of Co, 2H-MoS_2_@Co_3_S_4_ shows suitable E_ads_, enabling the changed adsorption configuration of oxygen-containing intermediates to efficiently adsorb and desorb the discharge product. Nevertheless, the overall electrical conductivity of 2H-MoS_2_@Co_3_S_4_ is poor due to the wide bandgap of 2H-MoS_2_. Moreover, 1T/2H-MCS could completely maintain dodecahedral hollow structure from ZIF-67 and be decorated with MoS_2_ nanosheets. This unique 3D architecture can effectively impede the agglomeration and restack of MoS_2_, exposing more active sites and promoting mass diffusion for faster reaction kinetics. Together with these synergistic effects, 1T/2H-MCS cathodes exhibit significantly improved electrochemical activities for both OER and ORR, including a discharge specific capacity of 16,875 mAh g^−1^ at 0.5 mA cm^−2^ with a capacity retention of up to 96% and a steady cycle life of 375 cycles at 0.5 mA cm^−2^ with a cutoff specific capacity of 1 mAh cm^−2^, and these data demonstrate their practical application potential for next-generation high energy–density power sources.

## Experimental Section

### Synthesis of ZIF-67

The typical reaction process for the preparation of ZIF-67 template is as follows. 3 mmol cobalt nitrate hexahydrate and 12 mmol 2-methylimidazole were dissolved in 30  and 10 mL of methanol , respectively, which were then mixed and aged at room temperature for 24 h. After complete centrifugal washing with methanol, ZIF-67 product was dried at 80 °C overnight.

### Synthesis of 1T/2H-MoS_2_@Co_3_S_4_ (1T/2H-MCS)

50 mg of as-prepared ZIF-67 templates were added into 10 mL ethanol under sonication for 10 min. Consequently, 10 mL aqueous solution containing Na_2_MoO_4_⋅2H_2_O (0.033 M) and thioacetamide (TAA, 0.1 M) was added slowly into the above solution under sonication for another 10 min. Afterward, the uniform mixture was placed into a 40-mL Teflon-lined stainless-steel autoclave, which was maintained at 120 °C for 4 h and then raised to 200 °C for 8 h. After being cooled down naturally to room temperature, final product was centrifuged and washed with ethanol and deionized water, followed by drying at 80 °C overnight.

### Material Characterizations

X-ray diffraction (XRD, Rigaku D/Max-r B) was used to characterize the crystal phases. Field-emission scanning electron microscopy (FESEM, JSM-7610F) and transmission electron microscopy (TEM, JEOL JEM-F200) with the acceleration voltage of 200 kV were applied to display the microstructures. Electrical conductivity was measured at room temperature by a ST-2258A digital four-point probe test system. Before the measurements, sample powders were compressed into a wafer with a thickness of 0.2 mm and a diameter of 13 mm by an oil pressure machine under a pressure of 30 MPa. Thickness of the nanosheets was measured by using an atomic force microscope (AFM) on a Bruker DI MultiMode-8 system. X-ray photoelectron spectroscopy (XPS, ESCALAB 250) was conducted to investigate surface chemical states. Raman spectra (LabRAM HR Evolution) with a 532-nm laser were used to further detect the material structures. Electron paramagnetic resonance (EPR, EMXnano) tests were performed at 300 K to confirm the defects. N_2_ adsorption–desorption measurements (ASAP 2020) were implemented to detect the surface areas and pore sizes. Inductively coupled plasma spectrometer (ICP-OES, Agilent 5110) was used to determine element contents. The gas evolution rates of O_2_, H_2_O, and CO_2_ were tested by in situ DEMS (i-DEMS 100). X-ray absorption fine structure (XAFS) spectra of Co K-edge were collected in transmission mode on a commercial Laboratory-Based XAFS spectrometer (Table XAFS-500A, Specreation Instruments Co., Ltd.). An X-ray tube was used to generate X-ray, and the voltage and current were set to 20 kV and 20 mA. The R250-mm Rowland circle was used to provide monochromatized X-ray beam.

### Electrochemical Measurements

LOBs were assembled using CR 2032 coin-type cells with holes in their positive top covers for electrochemical tests. Electrolyte was composed of 1 M lithium bis(trifluoromethanesulfonyl)imide dissolved in trimethylene glycol dimethyl ether (LiTFSI/TEGDME). Active material, Ketjen black, and polytetrafluoroethylene (PTFE) polymer binder were mixed in 5 mL isopropanol solvent with a mass ratio of 4:4:2 to obtain the slurry, which was coated on the carbon paper on a heating base at 80 °C to fabricate the cathode. Lithium foil and glass fiber membrane were served as anode and separator, respectively. LOBs were assembled in an argon-filled glove box (H_2_O < 0.1 ppm, O_2_ < 0.1 ppm) and placed in a chamber purged with O_2_ to investigate galvanostatic discharge/charge performance on a multichannel battery system (LAND CT 2001A). Cyclic voltammetry (CV) testing at 0.15 mV s^−1^ within the potential range of 2.35–4.50 V (vs Li/Li^+^) as well as electrochemical impedance spectroscopy (EIS) measurement with a limited frequency range of 10^5^ to 10^–2^ Hz and a 10 mV oscillation amplitude was carried out on the electrochemical workstation (CHI760E).

In a pouch-type LOB, lithium foil (4 × 4 cm^2^), 1T/2H-MCS-coated carbon cloth (4 × 4 cm^2^), and 1 M LiTFSI/TEGDME-soaked glass fiber membrane (4.5 × 4.5 cm^2^) were, respectively, used as the anode, the cathode, and the separator, which were encapsulated with perforated aluminum plastic film and sealed by a heat press sealer. The mass loading of 1T/2H-MCS is 16 mg for each cathode.

### Theoretical Calculations

First-principles computations were employed by the Vienna Ab initio Simulation Package (VASP) based on DFT. The exchange–correlation energy of electrons was described using generalized gradient approximation (GGA) with the Perdew–Burke–Ernzerhof (PBE) parametrization. The core valence interaction was performed using the projector augmented wave (PAW) method. The cutoff energy was 450 eV and the k-point mesh was set to be 1 × 1 × 1 for all slab calculations. The energy convergence criteria were set to 10^–5^ eV. A vacuum layer of 15 Å along the z-axis was applied to all slabs to avoid the periodic structural interactions. The maximum Hellmann–Feynman stress on each atom was set to be less than 0.02 eV Å^−1^. All atoms were fully relaxed for the heterojunction structure optimizations, while the lower four layers of atoms were fixed for the structure optimization of all the catalytic intermediates.

## Results and Discussion

### Theoretical Prediction on Double Complementary Effect in 1T/2H-MoS_2_@Co_3_S_4_

**Fig. 1:**
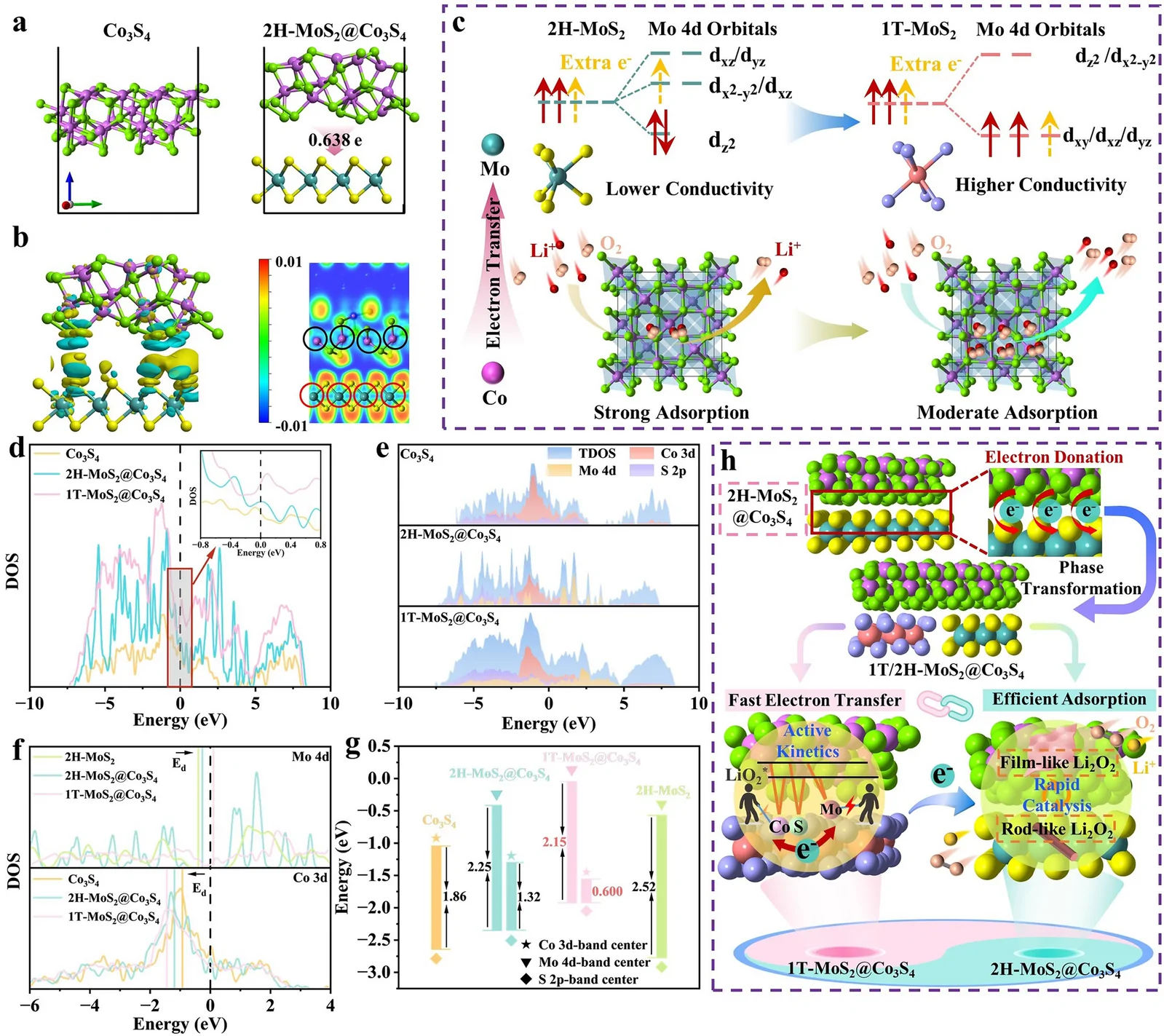
**a** Bader charge transfer, **b** ELF, and charge density difference of 2H-MoS_2_@Co_3_S_4_, in which charge accumulation and depletion are displayed by yellow and cyan, respectively. **c** Schematic diagram of proposed electron transfer processes between Co_3_S_4_ and 2H-MoS_2_. **d** TDOS and **e** PDOS of different samples. **f, g** *d*-band and p-band centers of Co 3*d*, Mo 4*d*, and S 2*p*. **h** Schematic diagram of double heterojunction effects on 1T/2H-MCS for LOBs

DFT calculations were conducted to investigate the phase transition mechanisms of MoS_2_ after combined with Co_3_S_4_, and Fig. S1 displays the optimal structural models of the Co_3_S_4_, 2H-MoS_2_, 2H-MoS_2_@Co_3_S_4_, and 1T-MoS_2_@Co_3_S_4_ heterostructures. In our previous research, DFT calculations of 2H-MoS_2_ have been carried out by the same way as in this work [36]. By Bader charge analysis, it is revealed that the electron transfer occurred from Co_3_S_4_ into 2H-MoS_2_ with an amount of 0.638 e, inducing phase transition from 2H-MoS_2_ to 1T-MoS_2_ (Figs. 1a and S2). Charge density difference and electron localization function (ELF) further prove the electron transfer from Co_3_S_4_ to 2H-MoS_2_ on heterogeneous interfaces, as shown in Fig. 1b. Therefore, the electron configuration of Mo 4*d* orbitals was rearranged. Based on crystal-field theory, Mo 4*d* orbitals of trigonal prismatically coordinated 2H-MoS_2_ cleavage into three energy levels: d_z2_ (a_1_′), d_x2-y2_/d_xy_ (e′), and d_xz_/d_yz_ (e′′) orbitals. Two Mo *d* electrons could completely be filled to the lower-lying a_1_′ orbital, endowing the 2H-MoS_2_ semiconducting characteristic with high structural stability. In contrast, Mo 4*d* orbitals of octahedrally coordinated 1T-MoS_2_ cleavage into d_xy_/d_xz_/d_yz_ (t_2g_) and d_z2_/d_x2-y2_ (e_g_) orbitals. Because of the incomplete occupation of the lower-lying t_2g_ orbitals, 1T-MoS_2_ shows metallic electronic property but poor structural stability [37].

To explain the interaction between Mo ions and the adjacent Co ions after combination, the partial electron transfer process from Co to Mo ions through the S ligand is illustrated in terms of their electronic configuration, as given in Figs. S3 and S4. As mentioned above, the a_1_′ orbital of Mo^4+^ with 4*d*^2^ trigonal prismatically coordinated geometry is fully occupied. In this condition, the major electronic interaction between the bridging S^2−^ and Mo^4+^ is e^−^-e^−^ repulsion. Similarly, trivalent Co ions present the valence electron configuration of 3*d*^6^ and exhibit low-spin octahedral geometry with fully occupied t_2g_ orbitals. After formation of heterostructures, the *d* electrons of Co 3*d* move to Mo 4*d*. Due to the relative higher electronegativity of Mo ions, it thus can be claimed that the e^−^–e^−^ repulsion of Co–S bonds aided in intensifying the π-donation interaction between Mo^4+^ and S^2−^, which trigger partial electron transfer from Co to Mo ions, accompanying the changes of coordination with surrounding S^2−^ [38]. As depicted in Fig. 1c, when 2H-MoS_2_ was combined with Co_3_S_4_, the extra electrons donated by Co_3_S_4_ firstly occupied the e′ orbitals of 2H-MoS_2_, destabilizing the structure and inducing the Mo 4*d* orbital reorganization to generate MoS_2_ transformation from 2H to 1T phase. As a result, the intrinsic electrical conductivity of MoS_2_ was largely improved, which could serve as conductive phase for Co_3_S_4_. Total density of states (TDOS) around the Fermi level in Fig. 1d exhibits stronger metallic properties in 1T-MoS_2_@Co_3_S_4_. Particularly, the orbitals of Co 3*d*, S 2*p*, and Mo 4*d* in Fig. 1e show substantial overlaps, implying strong electron interaction among them to accelerate electron transfer in the Co–S–Mo channels. Furthermore, after the combination of MoS_2_ and Co_3_S_4_, the electron transferred from Co to Mo ions induced the downshift of the E_d_ of Co and the opposite trend of Mo (Fig. 1f), respectively, narrowing the energy differences between Co 3*d* and S 2*p* band centers and Mo 4*d* and S 2*p* band centers and thereby leading to the strong hybridization among Co, S, and Mo (Fig. 1g). The strong hybridization could improve the covalency Co–S–Mo bonds, which can directly facilitate the electron transport between Co ions and oxygen adsorbates via Co–S–Mo channels and activate the Co sites during the ORR process. Besides, the E_ads_ of LiO_2_ on Co_3_S_4_ surfaces is extraordinarily strong, and it led to the high charge overpotentials, due to the fact that the decomposition of the discharge product needs to cross a higher energy barrier. However, when combined with 1T-MoS_2_, the E_ads_ of 1T-MoS_2_@Co_3_S_4_ could be reduced by modulated E_d_ of Co 3*d*, which will be discussed in the following parts.

Based on the above analysis, Fig. 1h systematically demonstrates the synergistic effects of double complementary heterojunctions derived from charge donation from Co to Mo ions in 1T/2H-MCS. Specifically, according to theoretical calculations, 2H-MoS_2_@Co_3_S_4_ possesses suitable E_ads_ to efficiently adsorb and desorb the discharge product, but the overall electrical conductivity is poor due to the wide bandgap of 2H-MoS_2_. On the contrary, owing to the increasing DOS at the Fermi energy level, 1T-MoS_2_@Co_3_S_4_ exhibits excellent electrical conductivity via fast electron transport channels of Co–S–Mo bonds, while low antibonding orbital occupancy is unfavorable for adsorption of oxygen-containing intermediates, resulting from the large bandgap between E_d_ of Co and Fermi level. Therefore, 1T/2H-MCS containing complementary double heterojunctions is expected to enhance the catalytic activities of Co_3_S_4_.

### Morphological and Structural Characterization

**Fig. 2:**
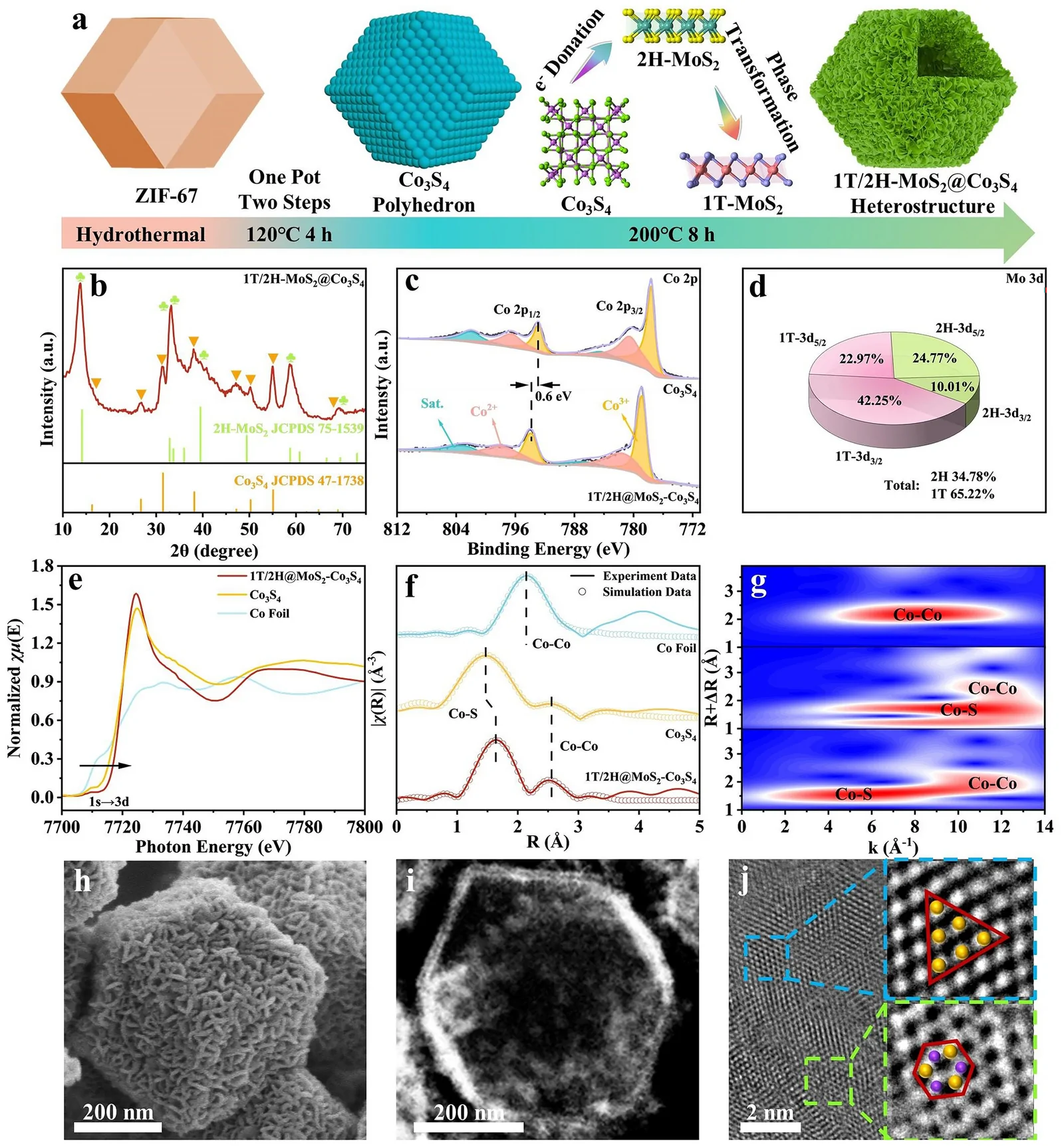
**a** Schematic illustration of synthetic process for 1T/2H-MCS with the proposed phase transformation mechanism. **b** XRD pattern, **c** high-resolution Co 2*p* XPS spectra, **e** Co K-edge XANES spectra, **f** Fourier transform of Co K-edge EXAFS spectra, and **g** wavelet transform analysis of different samples. **d** 1T and 2H phase ratios, **h** FESEM image, **i** HAADF image, and **j** HRTEM image with enlarged results from corresponding selected areas of 1T/2H-MCS

In a typical synthesis, ZIF-67 was applied as a template to obtain 1T/2H-MCS through a facile hydrothermal process (Fig. 2a), which could be divided into two steps. Under lower temperature, ZIF-67 was transformed into Co_3_S_4_ via a ligand exchange reaction between S_2_^−^ and 2-methylimidazole, still maintaining rhombic dodecahedral shape. After higher temperature, disordered MoS_2_ nanosheets grew on the surfaces of hollow Co_3_S_4_ polyhedrons to form 1T/2H-MCS. The digital photograph illustration of the synthesis path is given in Fig. S5.

To explore the crystalline structure and compositions of different samples, XRD, Raman, XPS, and EPR measurements were conducted. XRD analysis in Fig. 2b reveals that all characteristic peaks correspond to those of cubic Co_3_S_4_ (JCPDS 47-1738) and 2H-MoS_2_ (JCPDS 75-1539), proving that 1T/2H-MCS were successfully synthesized. Figures S6a, S7a, and S8a exhibit XRD patterns of the precursor, Co_3_S_4_, and 2H-MoS_2_, respectively. Raman data in Fig. S9 reveal that the peaks at 185 (A_g_), 298 (F_2g_), and 348 cm^−1^ (F_2g_) are related to Co_3_S_4_, while the peaks at 375 (E1 2g) and 399 cm^−1^ (A_g_) are associated with 2H-MoS_2_ [39–41]. Four distinctive peaks at 140 (J_1_), 193 (J_2_), 278 (E_1g_), and 333 cm^−1^ (J_3_) can be also observed, originating from the phonon modes of 1T-MoS_2_, and this demonstrates successful MoS_2_ phase conversion from 2H to 1T after combination [42].

XPS survey spectra in Fig. S10 overview the elements existing on different samples. To provide a direct comparison, the Co 2*p* XPS spectra of Co_3_S_4_ and 1T/2H-MCS are presented in Fig. 2e. Apparently, Co 2*p* XPS peak consists of two spin–orbit doublets, including Co 2*p*_3/2_ and Co 2*p*_1/2_ along with two satellites. Co^3+^ and Co^2+^ XPS peaks of Co_3_S_4_ position at 778.2/793.1 and 781.1/796.8 eV, whereas those of 1T/2H-MCS locate at 778.8/793.9 and 781.7/797.4 eV, respectively, indicating a binding energy red shift of 0.6 eV in 1T/2H-MCS relative to Co_3_S_4_. This XPS peak shift was attributed to changes in the oxidation state of the Co_3_S_4_ owing to MoS_2_ presence. Detailed comparisons of Co^2+^/Co^3+^ ratios in 1T/2H-MCS and Co_3_S_4_ are displayed in Fig. S11, showing the increased content of Co^3+^ from 48% to 65%. Co 2*p* XPS spectrum of 1T/2H-MCS displays higher oxidation states than that of Co_3_S_4_ owing to electron transfer at the interfaces of Co_3_S_4_ and 2H-MoS_2_, which is also confirmed by Bader charge results [43]. Co 2*p* XPS spectrum of 1T/2H-MCS displays higher oxidation states than that of Co_3_S_4_ owing to electron transfer at the interfaces of Co_3_S_4_ and 2H-MoS_2_ [43]. It is believed that high Mo electronegativity altered the Co electronic structure (σ*-orbital occupancy) by decreasing the Co electron density. Co σ*-orbital (e_g_) occupancy is close to unity, which can boost the intrinsic ORR and OER activities toward oxygen species by promoted covalence of Co–O bonds [44, 45]. Figure S12 shows the corresponding high-resolution Mo 3*d* XPS spectra of 2H-MoS_2_ and 1T/2H-MCS. Compared to Mo 3*d*_3/2_ (232.3 eV) and Mo 3*d*_5/2_ (229.2 eV) signals of pristine 2H phase, all the corresponding XPS peaks of 1T/2H-MCS blue-shift to lower binding energy at 231.9 and 228.8 eV. After fitting, the asymmetric signals can be divided into two peaks, and higher binding energy (labeled with green) and lower binding energy (labeled with pink), respectively, correspond to 2H and 1T MoS_2_ phases, which is well consistent with Raman results [46, 47]. In terms of the deconvolution result of Mo 3*d* XPS spectra, the ratio of 1T phase is over 65% in the MoS_2_ (Fig. 2d). It can be concluded the increased content of Co^3+^ could induce extra electron, occupying the e′ orbitals of 2H-MoS_2_ to realize Mo 4*d* orbital reorganization and MoS_2_ phase transformation from 2H to 1T. Accordingly, the high-resolution S 2*p* XPS spectra of 1T/2H-MCS in Fig. S13 are fitted into four peaks, which locate at 161.6/162.7 eV for 1T-MoS_2_, 162.1/163.2 eV for 2H-MoS_2_, and 163.7/164.9 eV for Co_3_S_4_. It is obvious that different shifts occurred for Co_3_S_4_ and 2H-MoS_2_, demonstrating that the construction of the heterostructures enabled electron transfer from Co_3_S_4_ to 2H-MoS_2_ through S-bridges. As shown in Fig. S14, EPR signal of 1T/2H-MCS (g = 2.003) corresponds to the unpaired Mo-S electrons on coordinatively unsaturated sulfur vacancy sites, which is stronger than those of Co_3_S_4_ and 2H-MoS_2_ [48, 49]. Compared with 2H-MoS_2_, the restriction growth of MoS_2_ by Co_3_S_4_ triggered more unpaired electrons on (002) lattice planes, exposing more unsaturated sulfur edges on the surfaces of 1T/2H-MCS, and this is in accordance with TEM results.

Brunauer–Emmett–Teller (BET) measurements based on N_2_ adsorption–desorption method were performed to investigate the specific surface areas and pore volumes. As shown in Fig. S15, the isotherms of 2H-MoS_2_, Co_3_S_4_, and 1T/2H-MCS conform to the H3-type hysteresis loop, implying the presence of mesoporous structures in different samples. Typically, the isotherms of 1T/2H-MCS with inconspicuous adsorption platform demonstrate that this mesoporous structure is irregular. Specific surface area of 1T/2H-MCS (47.54 m^2^ g^−1^) is larger than those of Co_3_S_4_ (22.74 m^2^ g^−1^) and 2H-MoS_2_ (11.34 m^2^ g^−1^). Compared with 2H-MoS_2_, it is supposed that S^2−^ can quickly react with Co^2+^ to form hollow Co_3_S_4_ with high surface area at low temperature. The growth of small MoS_2_ nanoflakes on the surfaces of Co_3_S_4_ at higher temperature could further enlarge the specific surface area. Moreover, the hollow Co_3_S_4_ structure endowed 1T/2H-MCS with a larger pore volume (0.21 cm^3^ g^−1^) than those of 2H-MoS_2_ (0.071 cm^3^ g^−1^) and Co_3_S_4_ (0.153 cm^3^ g^−1^). It is believed that large specific surface area is beneficial to expose more active sites, promoting the formation and decomposition of discharge product. Moreover, more space could be enabled for fast mass transfer and enough discharge product storage. More importantly, the abundant mesopores in 1T/2H-MCS could contribute to effective electrolyte penetration, enlarging the electrolyte–catalyst interface area to accelerate oxygen catalysis reactions [50].

To further detect electronic structure and coordination information of Co species in 1T/2H-MCS, X-ray absorption spectroscopy (XAS) analysis was conducted. X-ray absorption near-edge spectra (XANES) of Co K-edge in Fig. 2e show that the Co K-edge of 1T/2H-MCS shifts to a higher energy position than those of Co foil and Co_3_S_4_, standing for an increasing Co chemical state in 1T/2H-MCS. The higher Co valence state in 1T/2H-MCS is consistent with the theoretical prediction and XPS results, suggesting that electron transferred from Co to Mo sites when combined with 2H-MoS_2_ due to their electronegativity differences. Fourier transform (FT) k^3^-weighted EXAFS spectra of 1T/2H-MCS in Fig. 2f exhibit two apparent peaks located at 1.63 and 2.52 Å, which were ascribed to the Co–S and Co–Co contribution [51]. EXAFS spectra of Co K-edge were best fitted by including Co–S and Co–Co scattering path in fit model, as depicted in Table S1. Comparing 1T/2H-MCS with Co_3_S_4_, the first coordinative path of 1T/2H-MCS was expanded, which can be attributed to the asymmetric construction of Co–S–Mo after combining with 2H-MoS_2_. This coordination path can be better visualized in Fig. 2g by the maxima in the wavelet transform (WT) of the EXAFS, which is quite different compared to those of Co foil and Co_3_S_4_.

Morphology and structure information of these samples were captured by FESEM. As depicted in Fig. S6b, ZIF-67 exhibits a rhombic dodecahedral structure, and its surfaces are smooth without apparent wrinkles. Figure 2h presents the FESEM image of 1T/2H-MCS, which retained the typical dodecahedral rhombic shape of ZIF-67 and is decorated with random nanosheets with an average particle size of about 500 nm. For comparison, Figs. S7b and S8b show the FESEM images of Co_3_S_4_ and 2H-MoS_2_ samples, respectively, which display polyhedrons and large nanosheets with severe agglomeration. It is thus demonstrated that the ZIF-67 template can obviously restrict the layer-oriented growth of MoS_2_. In addition, AFM was used to detect the flake thickness of 1T/2H-MCS. The result in Fig. S16a reveals that the MoS_2_ nanoflakes formed by 6–7 monolayers, which is consisted with the following results of HRTEM images, and its corresponding 3D topography is offered in Fig. S16b. As shown in Figs. 2i and S17a, the high-angle annular dark-field (HAADF) and HRTEM images confirm that 1T/2H-MCS displays hollow structure containing Co_3_S_4_ nano-polyhedrons and outer MoS_2_ nanosheets. Selected area electron diffraction (SAED) pattern agrees well with the crystal plane reflection of Co_3_S_4_ and 2H-MoS_2_, verifying the successful synthesis of MoS_2_@Co_3_S_4_ heterostructures, as pictured in Fig. S18. From higher-magnification observation, the lattice fringe spacing of 0.284 nm corresponds to Co_3_S_4_ (311) planes, and there are some disordered regions generated by the lattice mismatch between Co_3_S_4_ and MoS_2_ (Fig. S17b). Interestingly, after formation of MoS_2_ and Co_3_S_4_ heterostructures, lattice structures of 1T-MoS_2_ in blue area and 2H-MoS_2_ in green area are observed simultaneously in Fig. 2j, further confirming the successful synthesis of 1T/2H-MCS. In Fig. S19, energy-dispersive X-ray (EDX) elemental mappings of 1T/2H-MCS manifest that Mo, Co, and S elements are evenly distributed throughout the entire architecture. Similarly, Figs. S20 and S21 display uniformly element distribution on Co_3_S_4_ and 2H-MoS_2_, respectively. Moreover, the element content results from EDX in Fig. S22 show that the molar ratio of Co_3_S_4_/MoS_2_ in 1T/2H-MCS is 53.8/46.2, which is close to the inductively coupled plasma optical emission spectroscopy (ICP-OES) result (52.1/47.9), as demonstrated in Table S2.

### Electrocatalytic Performance

**Fig. 3:**
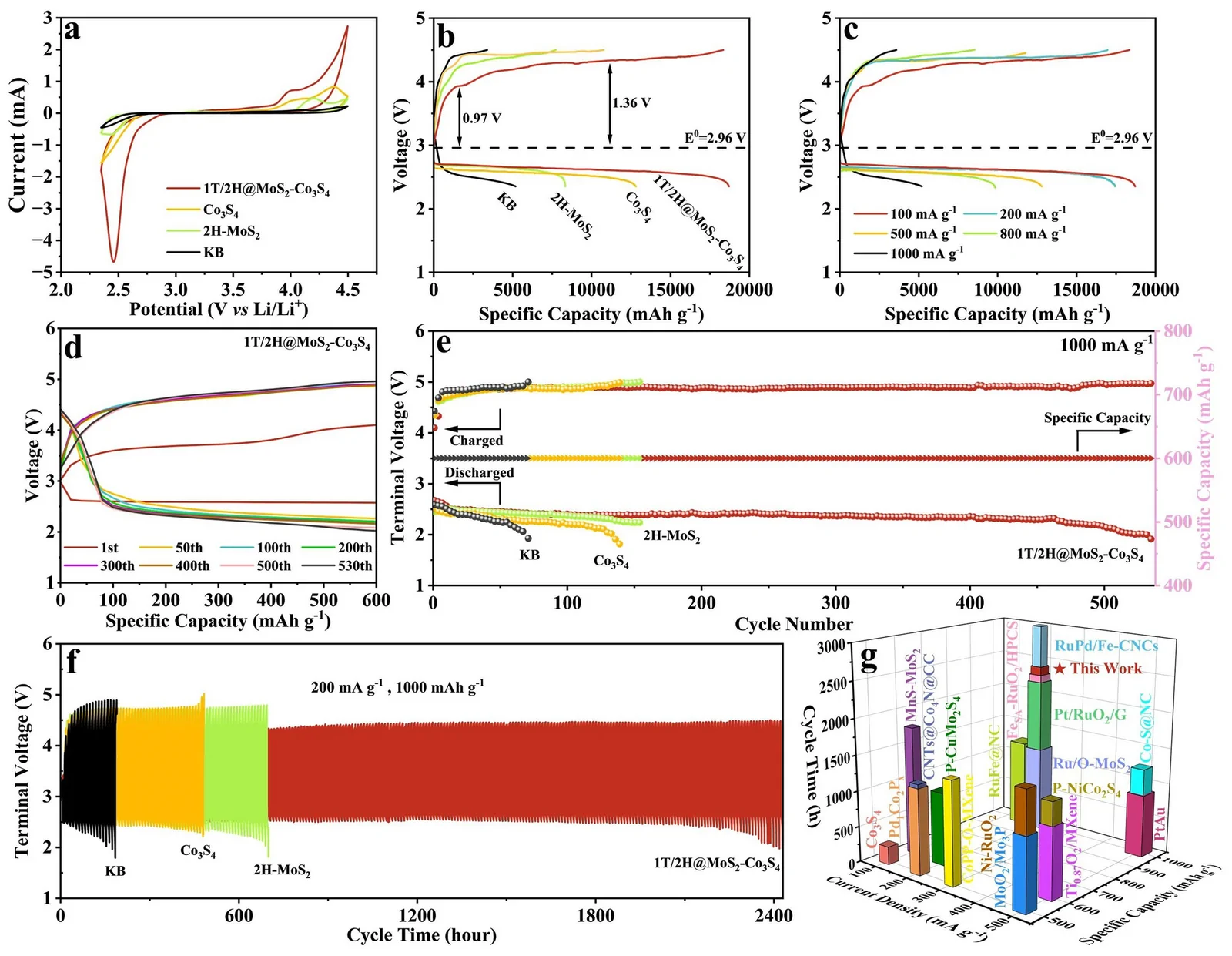
**a** CV curves, **b** initial discharge/charge curves at 100 mA g^−1^, **c** rate performance, as well as cycling duration **e** at 1000 mA g^−1^ under a specific capacity limit of 600 mAh g^−1^ with **d** selected charge–discharge profiles and **f** at 200 mA g^−1^ under a specific capacity limit of 1000 mAh g^−1^ of different cathodes. **g** Cycling stability comparison of 1T/2H-MCS cathode with representative and state-of-the-art cathodes containing Mo-, Co-, and noble metal-based compounds

Electrochemical tests were carried out to obtain insights into the ORR/OER catalytic activities of the prepared catalysts in LOBs. Figure 3a shows CV profiles of different cathodes within the voltage range of 2.35–4.50 V. It is found that 1T/2H-MCS cathode exhibits a higher onset potential for ORR (2.88 V) and a lower onset potential for OER (3.18 and 3.99 V), and its integral area is larger than those of other cathodes, implying better reaction kinetics and enhanced specific capacities [52]. During the cathodic scan, two small redox peaks during the ORR/OER processes demonstrate the stepwise formation/decomposition of intermediate LiO_2_, indicating the fast ORR/OER kinetics of 1T/2H-MCS cathode. Figure 3b displays the initial galvanostatic discharge/charge performance for different cathodes at 100 mA g^−1^. It is traced that 1T/2H-MCS cathode shows the highest discharge/charge specific capacities of 18,721/18,313 mAh g^−1^, whereas those of the Co_3_S_4_, 2H-MoS_2_, and KB cathodes are 12,823/10,775, 8346/7744, and 5,195/3,394 mAh g^−1^, respectively. Figure S23 shows the first discharge/recharge differential plots of 1T/2H-MCS cathode. The obvious peak corresponds to ORR voltage plateau at first discharging, while the two main peaks during OER process mean that charging reactions went through with two plateaus. It is apparent that 1T/2H-MCS cathode presents superior discharge/charge capacities with highest Coulombic efficiency and lowest overpotentials, agreeing well with CV results.

In Fig. 3c, the discharge/charge polarization of 1T/2H-MCS cathode gradually increased, and it still delivers impressive discharge specific capacities of 17,452, 12,804, 9,852, and 5,203 mAh g^−1^ at current densities of 200, 500, 800, and 1,000 mA g^−1^, respectively, with corresponding Coulombic efficiencies of 97.2%, 91.3%, 86.7%, and 68.9%. In addition, the rate performance of different cathodes was also evaluated by terminal discharge/charge voltage fluctuations at various current densities in Fig. S24. As current density increased (100, 200, 400, 800, and 1000 mA g^−1^), the discharge and charge terminal voltages of the 1T/2H-MCS cathode only show slight variations of 0.20 and 0.24 V, respectively. After the current density was set to a primitive value, the discharge/charge terminal voltages exhibit highly reversible recovery, further manifesting the excellent rate reliability of 1T/2H-MCS cathode. The resistivity value of 1T/2H-MCS was measured as 82 Ω cm by using four-point probe techniques, which is much lower than that of 2H-MoS_2_ (7.68 × 10^4^ Ω cm) and Co_3_S_4_ (1.86 × 10^3^ Ω cm) [53, 54]. It is believed that by the formation of 1T-MoS_2_ with the fast charge transfer channels of Co–S–Mo at the interfaces, the electronic conductivity of the 1T/2H-MCS cathode could dramatically increase, which enabled fast charge transfer and electrochemical performance improvement at high current densities.

Long-term cycling performance of different cathodes in LOBs was investigated. Figure S25 illustrates that 1T/2H-MCS cathode can stably work for 344 cycles, whereas the 2H-MoS_2_, Co_3_S_4_, and KB cathodes gradually decayed after 176, 149, and 60 cycles with a cutoff specific capacity of 600 mAh g^−1^ at 500 mA g^−1^. It can also be seen in Fig. 3d–f that 1T/2H-MCS cathodes display an impressive cycle duration of 535 cycles at a high current density of 1000 mA g^−1^ and an ultralong cycle life of 2420 h with a fixed specific capacity of 1,000 mAh g^−1^. Figure 3g and Table S3 show battery performance comparison of the representative and state-of-the-art cathodes containing Mo-, Co-, and noble metal-based compounds with the result in this work. It is worth noting that the cycling stability of 1T/2H-MCS cathode exhibits comparable performance and even outperforms some of noble metal-based counterparts. These improved electrocatalytic activities are attributed to the phase transformation of MoS_2_ from 2H to 1T by electron transfer, delivering a higher electrical conductivity, while the 2H phase can act as a stabilizer to keep the 1T phase structure stable during cycling. Meanwhile, the Co ions in Co_3_S_4_ with modulated e_g_ orbital occupancy after the electron transfer could promote the ORR/OER kinetics. In addition, after combining with 2H-MoS_2_, the E_d_ of Co slightly moved away from the Fermi level, which optimized the adsorption ability for the discharge product and further improved the battery performance.

### Practical Application in Pouch-Type Batteries

**Fig. 4:**
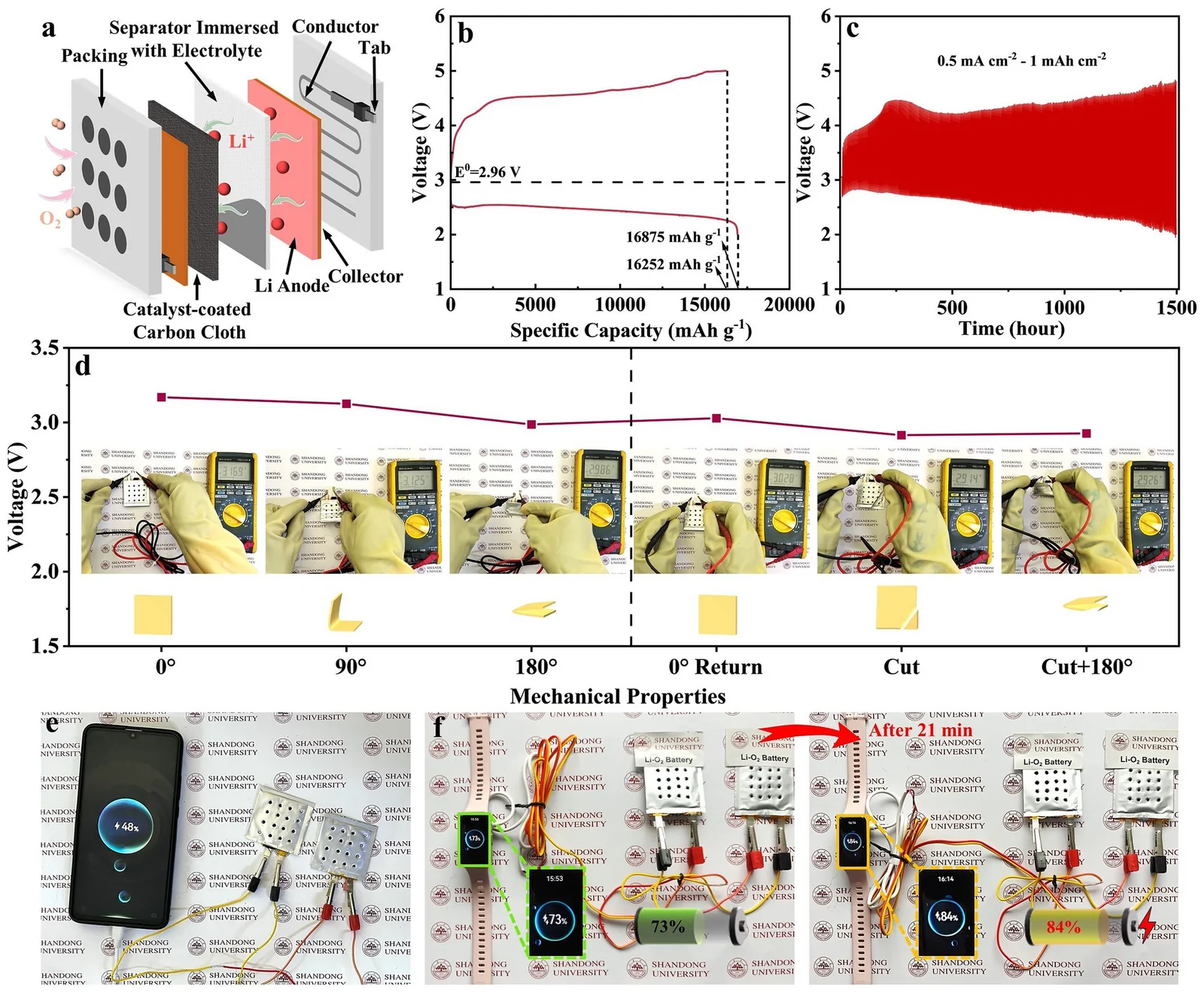
**a** Schematic illustration of the configuration, **b** initial discharge/charge performance, **c** cycling duration at 600 mA g^−1^ under a specific capacity limit of 1800 mAh g^−1^, **d** open-circuit voltages of different deformation states, and **e, f** practical applications of pouch-type LOBs

It is demonstrated in Fig. 4a that the flexible pouch-type LOB was constructed. As shown in Fig. 4b, it can obtain discharge/charge specific capacities of 16,875/16252 mAh g^−1^ and a high capacity retention of 96%, indicating excellent ORR/OER kinetics of 1T/2H-MCS cathode. Impressively, it could be continuously operated for 375 cycles with a limited specific capacity of 1 mAh cm^−2^ at 0.5 mA cm^−2^, revealing a considerable cycling stability, as offered in Fig. 4c. To clearly demonstrate battery electrochemical stability, the open-circuit voltages of the LOBs with 1T/2H-MCS cathodes were tested under different bending angles (Fig. 4d), displaying almost no change even battery corner was cut and was under 180° bent at the same time. It is also demonstrated that two pouch-type LOBs could be connected in series with the open-circuit voltage of 5.84 V (Fig. S26). As illustrated in Fig. 4e, the smartphone can be successfully charged using the above batteries, which can also charge a smartwatch with its power increasing from 73% to 84% in 21 min (Fig. 4f). It is suggested that high energy density and durability of the pouch-type LOBs with 1T/2H-MCS cathodes are highly beneficial for supporting next-generation electronic devices.

### Electrochemical Analysis

**Fig. 5:**
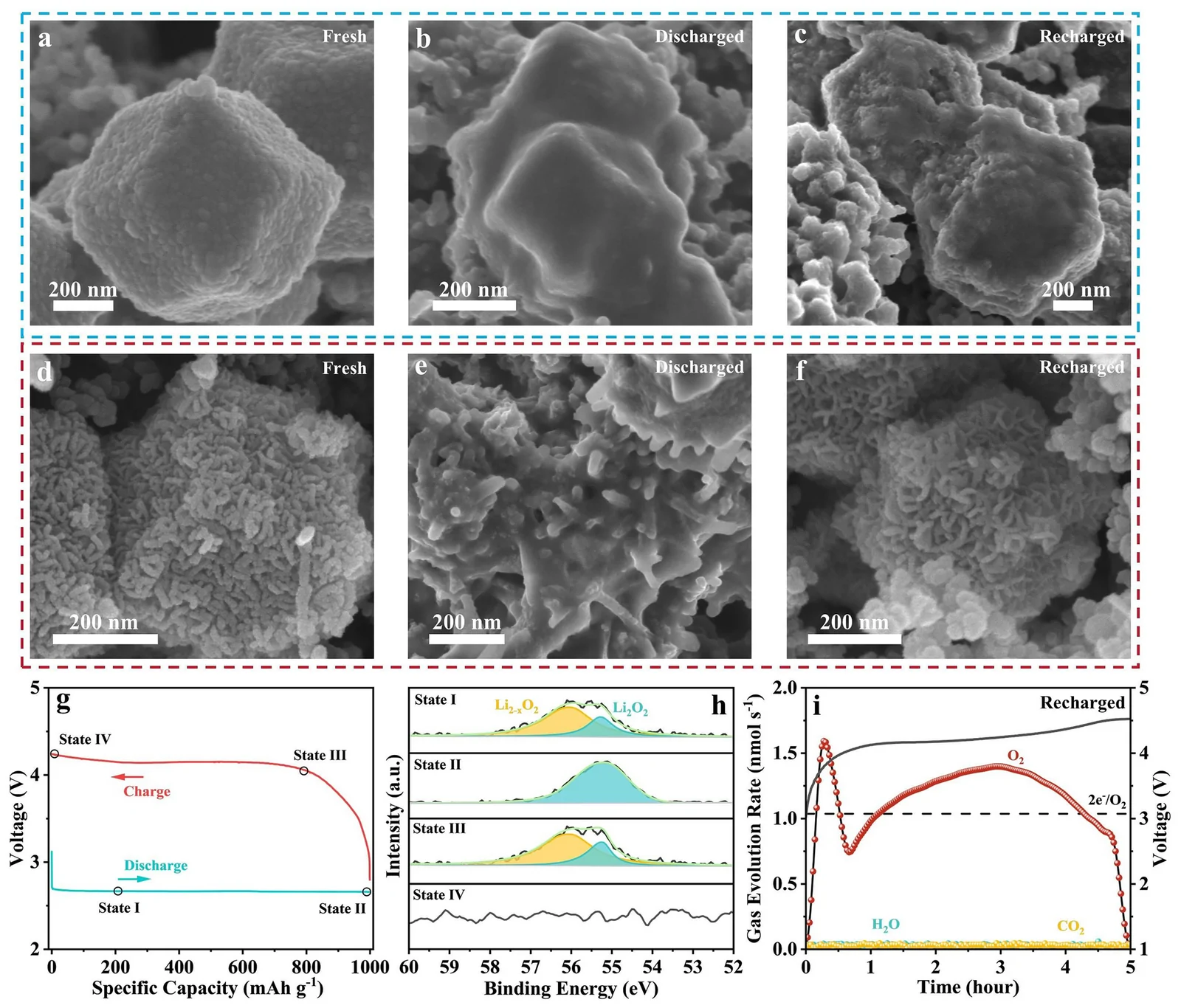
FESEM images of **a–c** Co_3_S_4_ and **d–f** 1T/2H-MCS cathodes at different states. **g** Initial discharge/charge curves at 200 mA g^−1^ under a cutoff specific capacity of 1000 mAh g^−1^, **h** high-resolution Li 1*s* XPS spectra at different states, and **i** in situ DEMS profiles with the charge curve at 200 mA g^−1^ of 1T/2H-MCS cathodes

To investigate the morphology and structural evolution of discharge product on the different cathodes, ex situ XRD, ex situ FESEM, ex situ XPS, and in situ differential electrochemical mass spectrometry (DEMS) analysis were applied to characterize the reaction processes at different states during discharge/charge processes. Ex situ XRD results show that the main discharge product on 1T/2H-MCS cathode is Li_2_O_2_ with poor crystallinity (Fig. S27), which disappeared after recharging. Even after 100 cycles, Li_2_O_2_ was nearly decomposed to expose catalyst surfaces again, demonstrating negligible accumulation of the discharge product and efficient catalysis activities of 1T/2H-MCS. Similar conclusion can also be drawn by Raman spectra in Fig. S28, and it is demonstrated that the side reactions were significantly inhibited, and the Li_2_O_2_ was fully decomposed [55]. Interestingly, the peaks of 1T-MoS_2_ were detected at different stages during cycling, indicating improved stability. According to previous studied, the existence of 2H-MoS_2_ can contribute to stabilizing 1T-MoS_2_, avoiding restacking as well as transformation into the 2H phase [35]. Furthermore, the morphology evolution of the 1T/2H-MCS, Co_3_S_4_, and 2H-MoS_2_ cathodes at different stages during 1st discharging/charging was observed from ex situ FESEM images. It is apparent that the dense film-like discharge product was adhered to the surfaces of Co_3_S_4_ cathodes, as shown in Fig. 5a, b, leading to cathode passivation with limited specific capacities [56]. However, since the E_ads_ of 2H-MoS_2_ for reaction intermediates is weak, the discharge product tends to form the rod-like Li_2_O_2_ through a solution-mediated pathway. It is noted that the poor catalytic activities of 2H-MoS_2_ as well as limited contact between it and the rod-like Li_2_O_2_ result in higher overpotentials and unsatisfactory cycle performance due to incomplete decomposition of discharge product, as can be seen in Fig. S29a, b. Interestingly, the film- and rod-like Li_2_O_2_ generated simultaneously on the surfaces of 1T/2H-MCS cathode, which were caused by moderate adsorption energy of active sites for reaction intermediates. Actually, the discharge product on 1T/2H-MCS cathode coexisted in two forms, allowing for more accommodation of discharge product and a significant increase in discharge specific capacity. Figure S30 displays the morphology of the discharge product for 1T/2H-MCS cathode after discharging to 1,000 mAh g^−1^. It is clear that the loose film-like and fine rod-like discharge product first formed, and it then grew into a uniform film and thick rod morphology with discharge capacity increase, which revealed the co-growth mechanism of the film- and rod-like Li_2_O_2_ on 1T/2H-MCS cathode. As shown in Fig. S31a, after 100th discharging, the film- and rod-like Li_2_O_2_ still could be observed on the surfaces of 1T/2H-MCS cathode, which is similar with that at 1st discharging, indicating the stability of the solution and surface dual reaction pathways on 1T/2H-MCS cathodes. Furthermore, the 1T/2H-MCS cathode could still maintain the original morphology after 100th recharging (Fig. S31b). In contrast, as shown in Figs. 5c, f and S29c, a large number of residues still remained on the surfaces of Co_3_S_4_ and 2H-MoS_2_ cathodes. They not only covered the active sites on the cathode surfaces, but also blocked the Li^+^/O_2_ transfer channels, thus ultimately leading to battery performance fails [57].

EIS plots of different cathodes were used to identify the intrinsic kinetics characteristic at different stages. As shown in Fig. S32, 1T/2H-MCS cathode presents smaller semicircles, and this implies a superior electrical conductivity for the phase transformation of MoS_2_ from 2H to 1T, which is well consistent with TDOS results in Fig. 1d. After discharging, it is clear that charge transfer resistance (*R*_ct_) increased from 21.9 to 85 Ω, which is still lower than those of Co_3_S_4_ and 2H-MoS_2_ cathodes due to the unique morphology evolution of the discharge product on cathode surfaces, promoting the charge transfer on 1T/2H-MCS cathodes. In addition, after 1st and 100th recharging, their *R*_ct_ shows insignificant changes compared to that of the fresh cathode. In contrast, a remarkable *R*_ct_ increment of Co_3_S_4_ and 2H-MoS_2_ cathodes can be detected after 1st discharging and charging, indicating that the dense film- and rod-like Li_2_O_2_/catalyst interfaces increased the charge transfer resistance and lead to obvious reaction polarization [58]. It is thus concluded that the 1T/2H-MCS cathodes show excellent electrical conductivity and moderate adsorption ability toward discharge product during cycling.

To further explore the chemical conditions of discharge product and reaction mechanisms in discharge/charge processes, ex situ high-resolution Li 1*s* XPS spectra of 1T/2H-MCS cathodes with selected pivotal states (Fig. 5g, h) at 200 mA g^−1^ were studied. It is worth noting that the primarily discharge product on 1T/2H-MCS cathode is Li_2-x_O_2_ (a low crystalline mixture of Li_2_O_2_ and LiO_2_) with the peak at 56.1 eV, and it transformed to Li_2_O_2_ during subsequent discharging, which is similar on Co_3_S_4_ and 2H-MoS_2_ cathodes [59]. However, signals of Li_2_CO_3_ appeared at 55.6 eV for Co_3_S_4_ and 2H-MoS_2_ cathodes. This indicates that side reactions occurred during the discharge process, and Li_2_CO_3_ remained when recharged to a limited specific capacity of 500 mAh g^−1^, which were accumulated during cycling and degraded battery performance, as evidenced in Figs. S33 and S34. Subsequently, in situ DEMS was conducted to determine the evolution of gas species in discharge/charge reactions. To calculate the electron-to-O_2_ (e^−^/O_2_) ratio, the intrinsic micro-electrochemical processes were evaluated using the following equations, as determined by the amount of e^−^ passed during charging and the integrating curves of the O_2_ consumption rate:

1 $${\text{Q }} = {\text{ I }} \times {\text{ t}}$$

2 $$\nu \, \left( {{\mathrm{e}}^{ - } } \right) \, = {\text{ Q }} \times {\text{ e}}^{ - }$$

3 $$\nu \, \left( {{\mathrm{O}}_{{2}} } \right) \, = {\text{ GasEvol}}.{\text{ Rate}}/{6}0 \, \times {\text{ N}}_{{\mathrm{A}}}$$

In situ DEMS results of different cathodes were obtained at 500 mA g^−1^ during charging, as provided in Figs. 5i, S35b, and S36b. It is clear that the e^−^/O_2_ ratio for 1T/2H-MCS was found to be close to 1.92, indicating the simultaneous decomposition of Li_2_O_2_ (2e^−^/O_2_) and LiO_2_ (e^−^/O_2_). In contrast, the values of Co_3_S_4_ and 2H-MoS_2_ are, respectively, 2.88 and 2.58, much higher than that for 1T/2H-MCS. This indicates incomplete decomposition of the discharge product and severe parasitic reactions, which is consistent with ex situ FESEM and ex situ XPS results [60]. Typically, O_2_ evolution plots for each cathode present typical “M” shape during charging, demonstrating stepwise decomposition of Li_2_O_2_, and an increased maximum O_2_ gas precipitation was presented at lower potential for 1T/2H-MCS cathode, which could be attributed to enhanced OER catalytic abilities [61]. Furthermore, it is apparent that an amount of CO_2_ gas evolved at the end of charging state for both Co_3_S_4_ and 2H-MoS_2_ cathodes, providing strong evidence that severe parasitic reactions occurred in recharge process to pose poor reversible specific capacity. During discharging, all curves are similar, and e^−^/O_2_ ratios are all close to the 2 for desired O_2_ reduction to Li_2_O_2_, as listed in Figs. S35a, S36a, and S37. These results demonstrate that the 1T/2H-MCS cathode could deliver moderate adsorption ability and significantly inhibit side reactions, thus exhibiting superior electrocatalytic activities for ORR/OER processes in LOBs.

To further elucidate the effect of double heterojunction effects in 1T/2H-MCS, samples of MoS_2_ mixed Co_3_S_4_ in the ratio of 1:1 (MoS_2_ Mixed Co_3_S_4_) were prepared. Since the DOS distributions of Co 3*d*, S 2*p*, and Mo 4*d* are mismatched, and it is hard to form chemical bonds between Co_3_S_4_ and MoS_2_ through simple mixing. In Fig. S38a, the ratio of Co^2+^/Co^3+^ in MoS_2_ Mixed Co_3_S_4_ is 46/54, which is similar to that in pure Co_3_S_4_, proving the limited charge donation from Co to Mo ions, and only 2H-MoS_2_ is present in MoS_2_ Mixed Co_3_S_4_, as shown in Fig. S38b. Consequently, MoS_2_ Mixed Co_3_S_4_ cathode shows unsatisfactory initial discharge/charge capacities of 11,037/8,400 mAh g^−1^ with poor Coulombic efficiency, as depicted in Fig. S39a. As for rate performance, discharge/charge terminal voltages decreased/increased obviously as the variation of current density (Fig. S39b). Furthermore, the EIS plots of MoS_2_ Mixed Co_3_S_4_ cathodes were collected at different stages in Fig. S40. The MoS_2_ Mixed Co_3_S_4_ cathode still displays a large *R*_ct_ of 169 Ω after recharging, indicating sluggish reaction kinetics during OER process. The above results confirm that the double heterojunction effects in 1T/2H-MCS play a critical role in enhancing electrochemical performance.

### Proposed Mechanisms by DFT Calculations

**Fig. 6:**
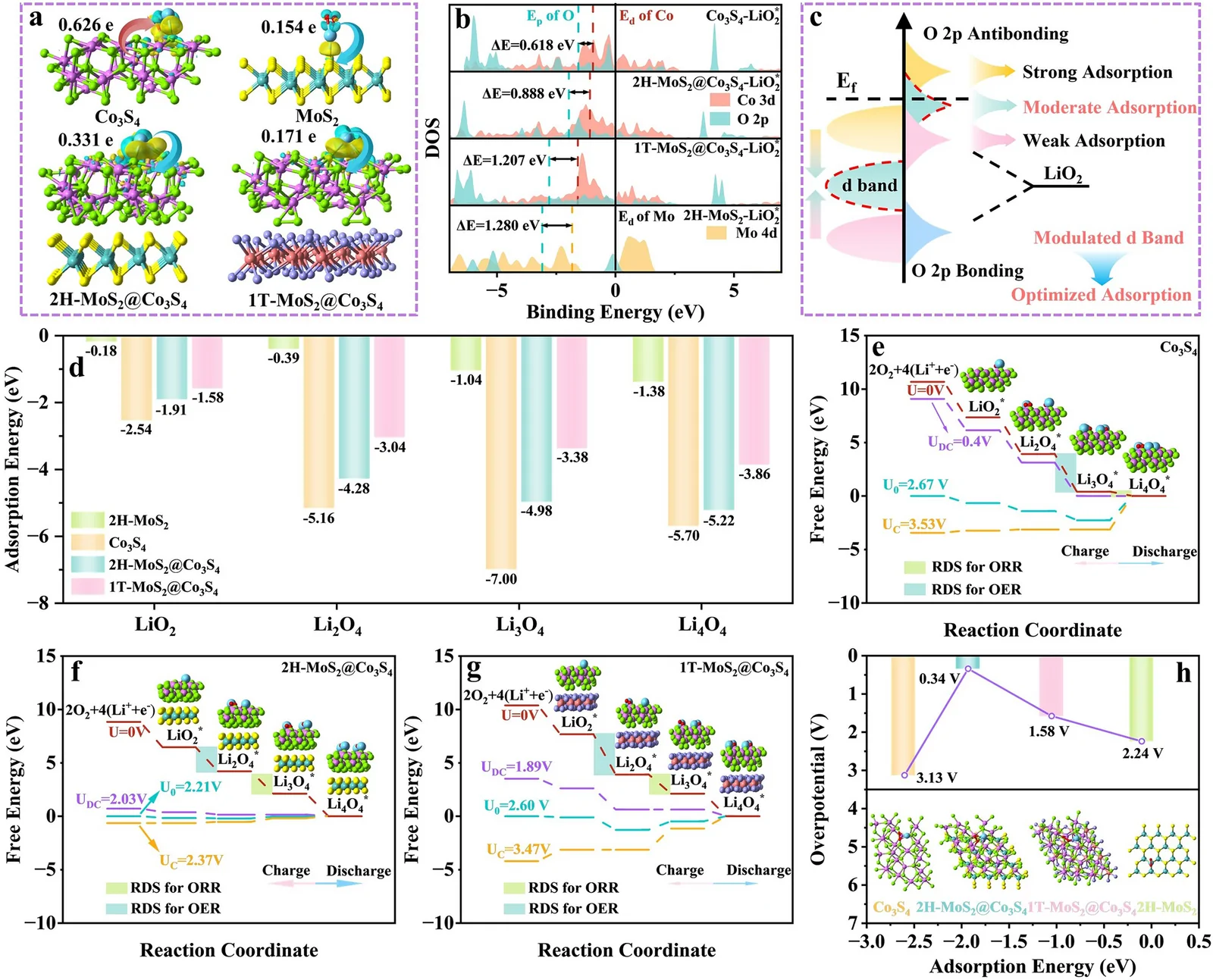
**a** Charge density differences with Bader charge transfer, **b** d-band and p-band centers of Co 3*d* and S 2*p* of different catalysts. **c** Schematic illustration of d-p orbital hybridization for 1T/2H-MCS. **d** Adsorption energy and the relationship between adsorption energy and overpotentials, **e–g** Calculated energy diagrams, and **h** relationship between adsorption energies and overpotentials of different catalysts

To further clarify the catalytic mechanisms, DFT calculations were carried out to simulate the formation/decomposition of the discharge product on the cathode catalysts. Figure S41 shows different optimized structures after adsorbing different oxygen species (LiO_2_, Li_2_O_4_, Li_3_O_4_, and Li_4_O_4_), and corresponding charge density difference plots and Bader charge transfer of different oxygen species on the surfaces of Co_3_S_4_, 2H-MoS_2_, 2H-MoS_2_@Co_3_S_4_, and 1T-MoS_2_@Co_3_S_4_ are shown in Figs. 6a and S42-S45. In those models, the yellow and blue areas denote the gained electrons and lost electrons, respectively. As for the adsorption models of 2H-MoS_2_, 2H-MoS_2_@Co_3_S_4_, and 1T-MoS_2_@Co_3_S_4_, the charge could transfer from oxygen-containing intermediates to catalysts, with more charge transferred to the active sites on 2H-MoS_2_@Co_3_S_4_, efficiently promoting desorption of LiO_2_ and thus greatly enhancing catalyst reaction kinetics [62]. While for the Co_3_S_4_, the charge transferred to oxygen-containing intermediates, which is unfavorable for the catalytic conversion and lead to increased overpotentials [63].

It is noted that covalency of metal–oxygen bonds serves as another essential descriptor for the electrocatalytic activities during the reaction processes, as it is closely linked to the interfacial electron transfer kinetics involved in oxygen redox reactions [64]. Typically, a modest increased covalency of metal–oxygen bonds tends to mitigate the electron transfer energy barrier. It can be found in Fig. 6b that PDOS shows a middle overlap degree between Co 3*d*- and O 2*p*-orbitals near the Fermi level in 2H-MoS_2_@Co_3_S_4_, compared to those in Co_3_S_4_ and 1T-MoS_2_@Co_3_S_4_, which demonstrates the moderate adsorption of LiO_2_ on the 2H-MoS_2_@Co_3_S_4_. Simultaneously, an increased energy gap between the Co 3*d* and O 2*p* band centers can be observed in Fig. 6b. The expanding of the energy difference between the Co 3d and O 2*p* band centers also manifests the weak hybridization between the Co 3*d* and O 2*p*, undermining the covalency of Co–O bonds. According to Sabatier principle [65], the strong covalency of Co–O bonds could result in the deactivation of active sites to increase the overpotentials. On the contrary, weak covalency of Co–O bonds could lead to unrestricted growth of Li_2_O_2_ and thus limit the contact between Li_2_O_2_ and cathode surfaces, deteriorating LOB performance. Besides, the overloaded adsorbed discharge product was prone to fall off the cathode, inducing discharge product loss at deep discharge state. Thus, it is concluded that 2H-MoS_2_@Co_3_S_4_ with moderate d-p orbital hybridization of LiO_2_ could exhibit abundant adsorption sites and superior OER/ORR kinetics. Furthermore, the 2H-MoS_2_@Co_3_S_4_ possesses optimized E_d_ value of Co with moderate Co–O antibonding orbital occupancy, which contributed to the optimal bonding interaction between Co sites and oxygen-containing intermediates for suitable oxygen adsorption and boosted electrocatalysis (Fig. 6c). Distinct differences between four models before and after adsorption of LiO_2_ can be traced in Fig. S46, indicating effective adsorption of 2H-MoS_2_@Co_3_S_4_ due to the optimized d-p orbital hybridization with LiO_2_. Detailed adsorption energy of the four catalysts for the reaction intermediates is clearly observed in Fig. 6d. It is widely accepted that the intrinsic adsorption energy (ΔE_ads_) of LiO_2_ intermediate on catalyst surfaces is a crucial factor in regulating the nucleation and growth mechanisms of Li_2_O_2_ during discharge process [66, 67]. Strong affinity of LiO_2_ on Co sites of Co_3_S_4_ promoted the accumulation of LiO_2_ with high local concentration and hence resulted in generation of dense film-like Li_2_O_2_ through surface growth pathway, which is unfavorable for mass diffusion and thus suppressed the subsequent Li_2_O_2_ formation. As for 1T-MoS_2_@Co_3_S_4_ and 2H-MoS_2_, due to their poor adsorption capability of LiO_2_, LiO_2_ tended to be dissolved in electrolyte and disproportionate to form Li_2_O_2_ nanorods. The oxidation of Li_2_O_2_ nanorods brought about high overpotentials because of poor interfacial contact between nanorods and catalytic active surfaces.

The TDOS data for Co_3_S_4_, 1T-MoS_2_@Co_3_S_4_, 2H-MoS_2_@Co_3_S_4_, and 2H-MoS_2_ before and after adsorptions of different reaction product are given in Fig. S47. TDOS result of 2H-MoS_2_@Co_3_S_4_ around the Fermi energy level exhibits a significant increase, and this represents enhanced electrical conductivity upon loading of the intermediates, which due to the optimized interaction between 2H-MoS_2_@Co_3_S_4_ and discharge product. However, opposite change for Co_3_S_4_ appears, suggesting that the insulated discharged product decayed the electrical conductivity of Co_3_S_4_ due to the strong adsorption ability. Phase diagram of the cathode reactions provides in-depth insights into the process of discharge product evolution, as depicted in Fig. S48. As for 2H-MoS_2_@Co_3_S_4_, LiO_2_ with a low ΔG formed spontaneously in the interval of 2.17–2.37 V. When the discharge voltage is below 2.17 V (the cross point between Li_2_O_4_ and Li_3_O_4_ line), Li_2_O_2_ with a thermodynamic advantage began nucleation. As the voltage dropped to 2.05 V, steady Li_4_O_4_ clusters generated. These results demonstrate that Li_2-x_O_2_ easily grew on 2H-MoS_2_@Co_3_S_4_ surfaces, which could change the nucleation route of Co_3_S_4_ after the formation of 1T/2H-MCS. To understand the configuration of oxygen-containing intermediates during the discharging process, O–O bond lengths, O–Li–O bond angles, and adsorption distances of oxygen-containing intermediates of four catalysts are compared in Figs. S49–S52. Due to the strongest/weakest d-p orbital hybridization of Co_3_S_4_ and MoS_2_, different oxygen-containing intermediates are all closest/farthest to/from the catalyst surfaces, which is in accordance with adsorption energy results. Furthermore, the bond lengths of O–O in oxygen-containing intermediates on 2H–MoS_2_@Co_3_S_4_ are in the middle of those on 1T-MoS_2_@Co_3_S_4_ and Co_3_S_4_, and the O–Li–O bond angles on 2H-MoS_2_@Co_3_S_4_ are twisted to a larger angle (the standard value is 45.6°), leading to a facile cleavage of the O–O bonds. Accordingly, heterojunctions of 2H-MoS_2_@Co_3_S_4_ could play an effective role in optimizing the adsorption of oxygen species and accelerating the O–O bond breakages.

Free energy calculations were used to determine the energy required for each reaction stage. OER/ORR overpotentials are defined as η_OER_ = U_C_-U_0_/η_ORR_ = U_0_-U_DC_. U_C_, U_DC_, and U_0,_ respectively, denote minimum charge, maximum discharge, and equilibrium potentials. 2H-MoS_2_@Co_3_S_4_ cathode exhibits the lowest η_OER_/η_ORR_ (0.16/0.18 V) among those of Co_3_S_4_ (0.86/2.27 V), 1T-MoS_2_@Co_3_S_4_ (1.17/0.81 V), and MoS_2_ (0.70/1.54 V) in Fig. 6f, revealing significantly promoted electrocatalytic activities of the 2H-MoS_2_@Co_3_S_4_ toward oxygen redox reactions in LOBs due to the moderate d-p orbital hybridization with oxygen-containing intermediates (Fig. 6e, g). Based on the above results, volcano-like relationship between binding energy of oxygen-containing intermediates with different catalysts and overpotentials can be established in Fig. 6h. Optimal binding energy of oxygen-containing intermediates on 2H-MoS_2_@Co_3_S_4_ is of great essence to improve its electrocatalytic activities, which is in conformity with the Sabatier principle. Particularly, owing to weak adsorption of oxygen-containing intermediates, the coverages of reactive oxygen species on 1T-MoS_2_@Co_3_S_4_ and 2H-MoS_2_ are relatively low and thus contributed less to oxygen electrocatalytic activities and electron transfer. On the contrary, excessive adsorption of reactive oxygen species on Co_3_S_4_ poisoned active sites and thus impeded reaction kinetics. Therefore, an appropriate binding energy of oxygen species on 2H-MoS_2_@Co_3_S_4_ could not only increase reactive oxygen species coverage, but also prevent poisoning effect caused by blockage of active sites, thereby beneficial for remarkable electrochemical performance of 1T/2H-MCS cathodes.

**Scheme 1:**
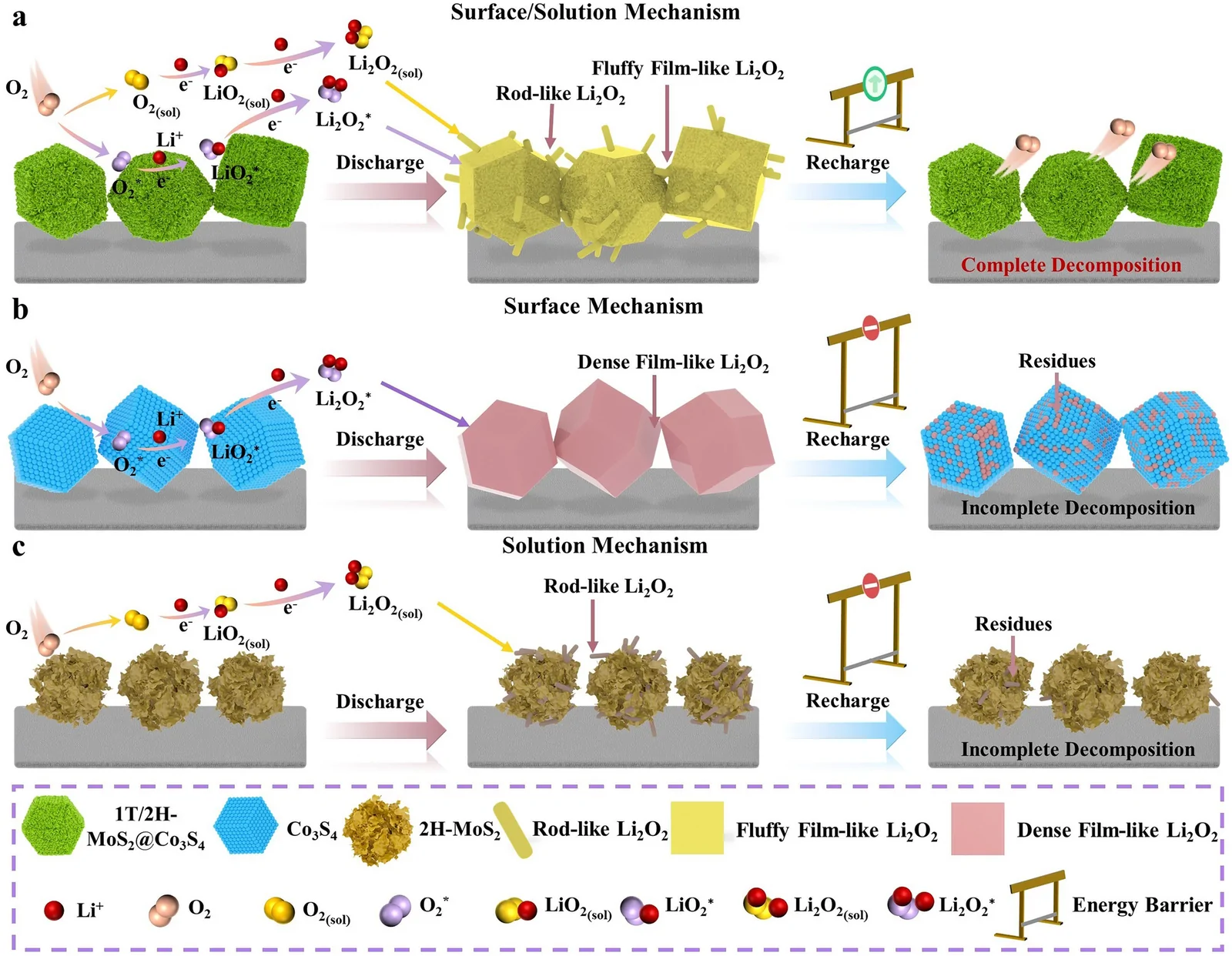
Proposed electrocatalytic mechanisms of **a** 1T/2H-MCS, **b** Co_3_S_4_, and **c** 2H-MoS_2_ cathodes during ORR/OER processes

Rational formation and decomposition pathways of the discharge product on different cathodes are elucidated in Scheme 1. It is proposed that intermediate product LiO_2_ first generated via a one-electron reduction reaction on the cathode surfaces (Eq. (4)). For the 2H-MoS_2_ cathode, due to extremely weak binding energy for LiO_2_, the intermediate was then dissolved in the electrolyte, defined as LiO_2(sol)_, which was further converted to the rod-like Li_2_O_2_ via solution growth pathway (Fig. S29b and Eq. (5)). During charging process, low catalytic activities, poor electrical conductivity, and limited contact with rod-like Li_2_O_2_ resulted in extremely low discharge specific capacities and unsatisfactory cycle performance of 2H-MoS_2_ cathodes. Inversely, the strong adsorption energy of LiO_2_* on the Co_3_S_4_ surfaces promoted the formation of dense film-like Li_2_O_2_ through surface growth pathway (Fig. 5b and Eq. (6)). They were closely adhered to the cathode surfaces to slow down the kinetic diffusion of electrons/ions on the cathode catalyst, which lowered the activation and led to high interfacial polarization. Fortunately, owing to moderate adsorption energy of 1T/2H-MCS for LiO_2_*, Li_2_O_2_ were grown on 1T/2H-MCS cathode via solution and surface dual pathways, forming two kinds of different morphologies (Fig. 5e). Enhanced catalytic activity of 1T/2H-MCS via the synergistic effects of double complementary heterojunctions from 1T-MoS_2_@Co_3_S_4_ and 2H-MoS_2_@Co_3_S_4_ changed the affinity between the catalyst surfaces and the reaction intermediates to affect LiO_2_ nucleation, successfully achieving the ideal Li_2_O_2_ morphologies.

4 $${\mathrm{O}}_{{2}} + {\text{ e}}^{ - } + {\text{ Li}}^{ + } \to {\mathrm{LiO}}_{{2}}$$

5 $${\mathrm{2LiO}}_{{{2}({\mathrm{sol}})}} \to {\mathrm{Li}}_{{2}} {\mathrm{O}}_{{2}} + {\text{ O}}_{{2}}$$

6 $${\mathrm{LiO}}_{{2}} * \, + {\text{ Li}}^{ + } + {\text{ e}}^{ - } \to {\mathrm{Li}}_{{2}} {\mathrm{O}}_{{2}} *$$

## Conclusions

In summary, ZIF-67 as a template was used to synthesize 1T/2H-MCS composites via one-pot hydrothermal method with two-step temperature-raising process, which served as efficient bifunctional electrocatalysts for LOBs. It is evident that 1T/2H-MCS cathodes display extraordinary electrochemical properties, including high reversible discharge/charge specific capacities of 18,721/18,500 mAh g^−1^ at 100 mA g^−1^ and ultralong cycle life of 2420 h at 100 mA g^−1^ under a cutoff specific capacity of 1000 mAh g^−1^. When applied in pouch-type LOBs, 1T/2H-MCS cathodes exhibit an impressive and promising potential for practical applications. Based on DFT calculations and experimental results, it is proposed that significantly improved OER/ORR performance of 1T/2H-MCS cathodes is owing to the complementary effects of double heterojunctions derived from the charge donation form Co to Mo ions. Firstly, 1T-MoS_2_@Co_3_S_4_ provided unblocked electron transport channels of Co–S–Mo bonds, well redistributing electrons within 1T/2H-MCS and hence significantly improving its interfacial electron transfer kinetics and electrocatalytic activities. Then, 2H-MoS_2_@Co_3_S_4_ possesses a moderate antibonding orbital occupancy when absorbed oxygen-containing intermediates, optimizing the morphology of discharge product and accelerating their decomposition by modulating the adsorption energy. Furthermore, 1T/2H-MCS holds a dodecahedral hollow structure inherited from ZIF-67 and is decorated with MoS_2_ nanosheets, which could restrict layer-oriented growth of MoS_2_ and increase specific surface area to expose abundant stable active sites. This complementary relationship provides guidance for designing efficient electrocatalysts with double heterojunctions for LOBs.

## Supplementary Information

Below is the link to the electronic supplementary material.

Supplementary file1 (DOCX 18687 KB)
