# Supplementary material for: Constructing Double Heterojunctions on 1T/2H-MoS2@Co3S4 Electrocatalysts for Regulating Li2O2 Formation in Lithium-Oxygen Batteries
Source: Nanomicro Lett. 2025 Sep 1;18:51. doi: 10.1007/s40820-025-01895-x (PMC12401821; doi:10.1007/s40820-025-01895-x)
Supplement: Supplementary file 1 — Supplementary file1 (DOCX 18687 KB) [file 40820_2025_1895_MOESM1_ESM.docx]

Supporting Information for

**Constructing Double Heterojunctions on 1T/2H-MoS_2_@Co_3_S_4_ Electrocatalysts for Regulating Li_2_O_2_ Formation in Lithium-Oxygen Batteries**

Yichuan Dou^1,4^†, Zhuang Liu^1,4^†, Lanling Zhao^2^*, Jian Zhang^3^, Fanpeng Meng^3^, Yao Liu^1,4^, Zidong Zhang^1,4^, Xingao Li^2^, Zheng Shang^2^, Lu Wang^2^ and Jun Wang^1,4^*

^1^ Key Laboratory for Liquid-Solid Structural Evolution and Processing of Materials (Ministry of Education), Shandong University, Jinan 250061, P. R. China

^2^ School of Physics, Shandong University, Jinan 250061, P. R. China

^3^ Shandong Guiyuan Advanced Ceramics Co., Ltd, Zibo 255020, P. R. China

^4^ Shandong Key Laboratory of Metamaterial and Electromagnetic Manipulation Technology, Jinan 250061, P. R. China

† Yichuan Dou and Zhuang Liu contributed equally to this work.

* Corresponding authors. E-mail: [jw707@sdu.edu.cn](mailto:jw707@sdu.edu.cn) (Jun Wang); [lanling@sdu.edu.cn](mailto:lanling@sdu.edu.cn) (Lanling Zhao)

**Supplementary Figures and Tables**

**
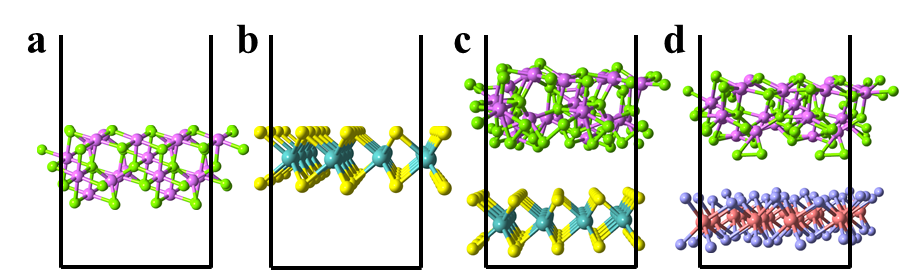
**

**Fig. S1** Optimized geometric structures of **a** Co_3_S_4_, **b** 2H-MoS_2_, **c** 2H-MoS_2_@Co_3_S_4_, and **d** 1T-MoS_2_@Co_3_S_4_

**
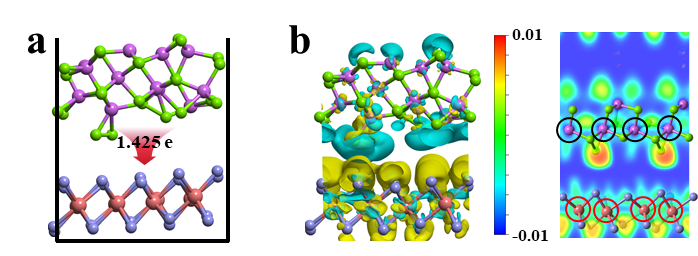
**

**Fig. S2 a** Bader charge transfer and **b** charge density difference with electron localization function (ELF) of 1T-MoS_2_@Co_3_S_4_. Charge accumulation and depletion are displayed by yellow and cyan, respectively

**
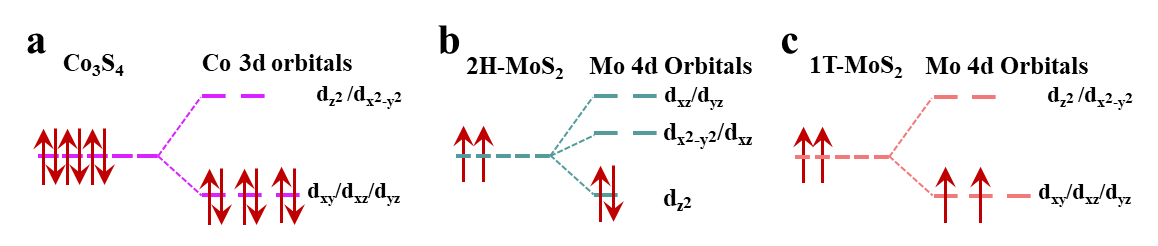
**

**Fig. S3 a** Co 3d-orbitals of Co_3_S_4_ and Mo 4d-orbitals of **b** 2H- and **c** 1T- MoS_2_

**
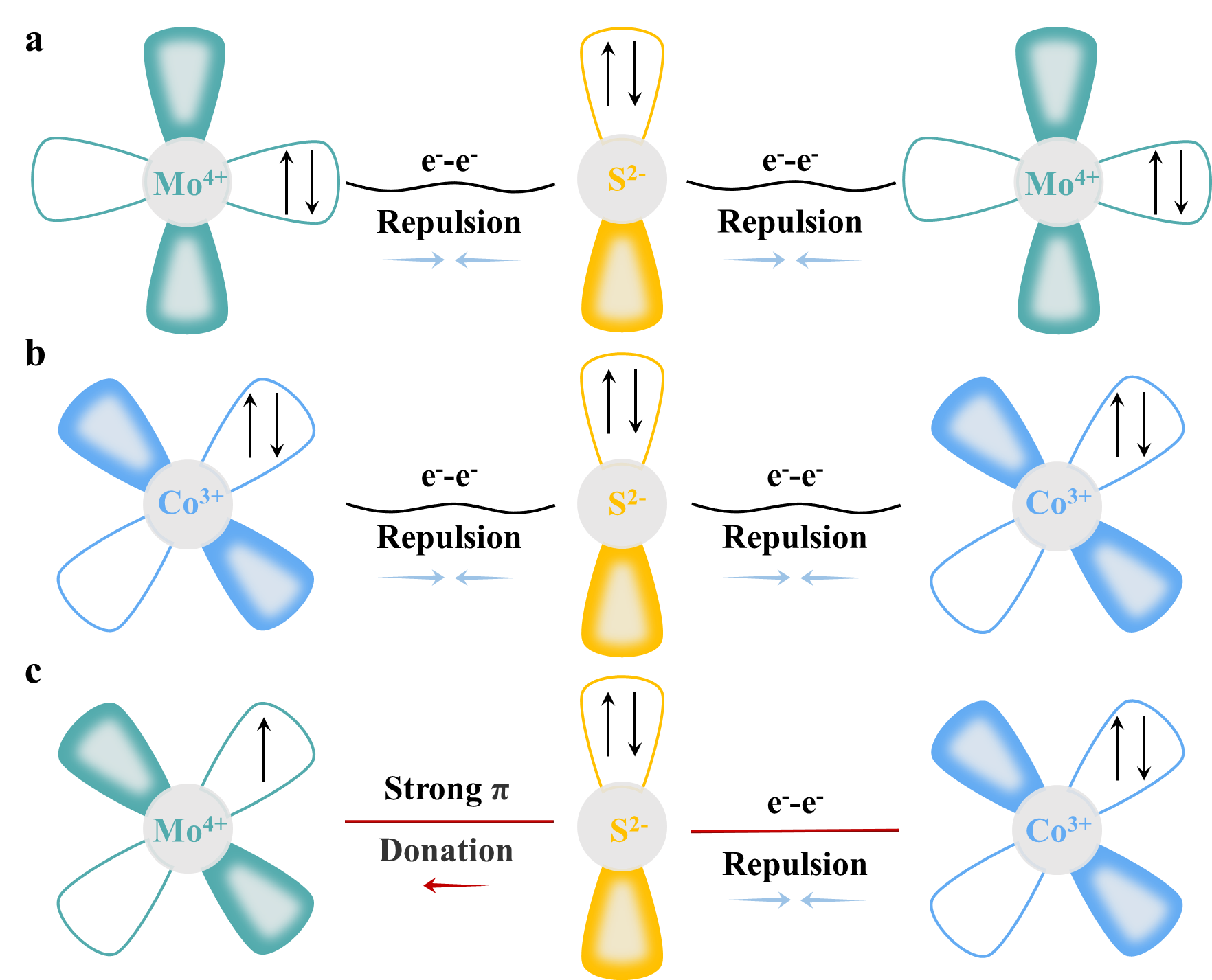
**

**Fig. S4** Schematic illustration of the electron couplings between Co, S, and Mo in **a** 2H-MoS_2_, **b** Co_3_S_4_, and **c** 1T/2H-MCS

**
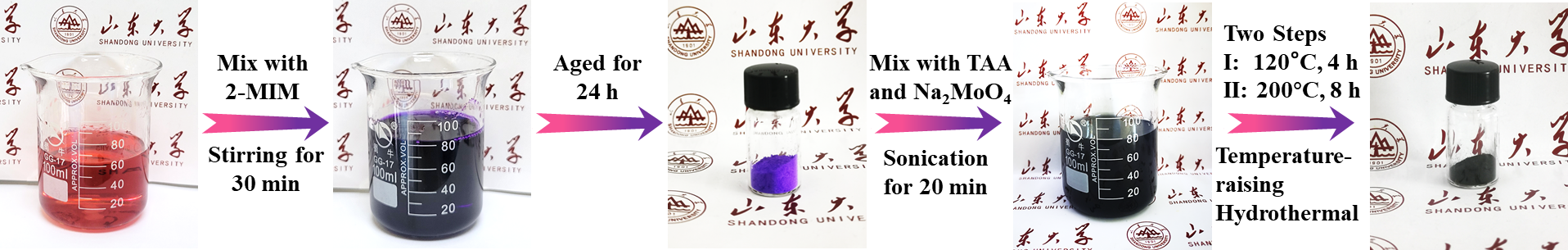
**

**Fig. S5** Digital photos of the products in the synthesis process

**
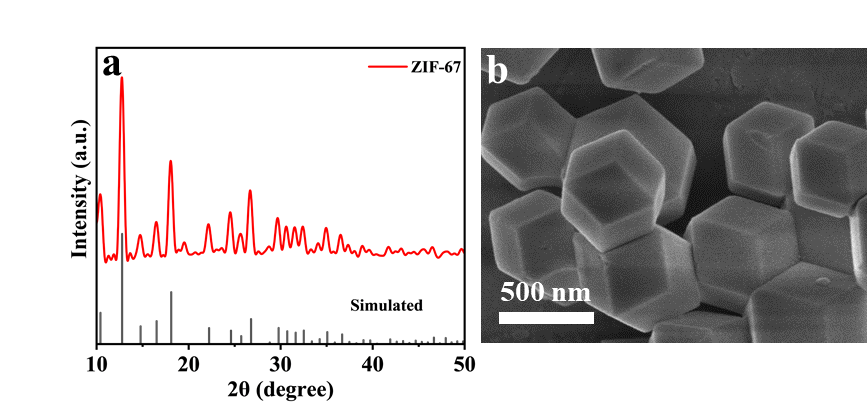
**

**Fig. S6** **a** XRD pattern and **b** FESEM image of ZIF-67

**
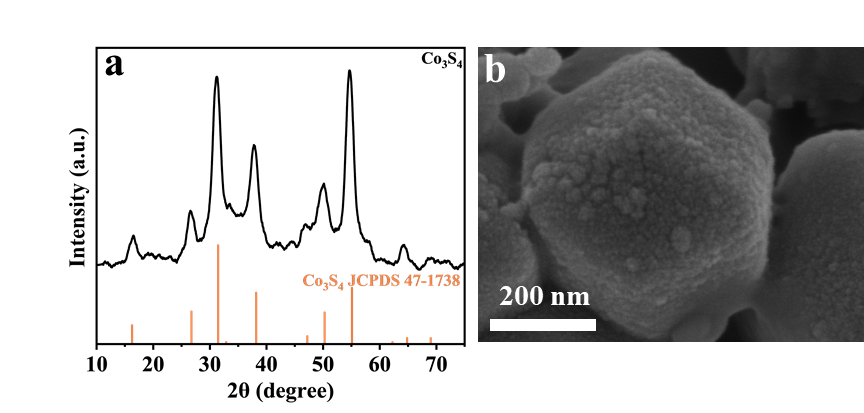
**

**Fig. S7** **a** XRD pattern and **b** FESEM image of Co_3_S_4_

**
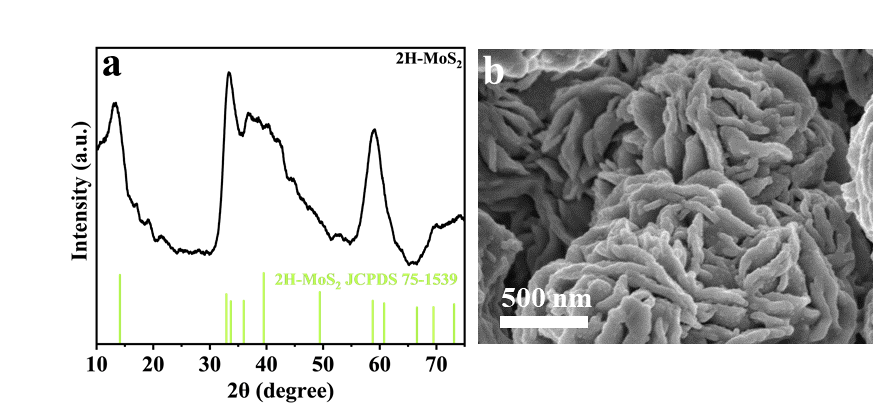
**

**Fig. S8** **a** XRD pattern and **b** FESEM image of 2H-MoS_2_

**
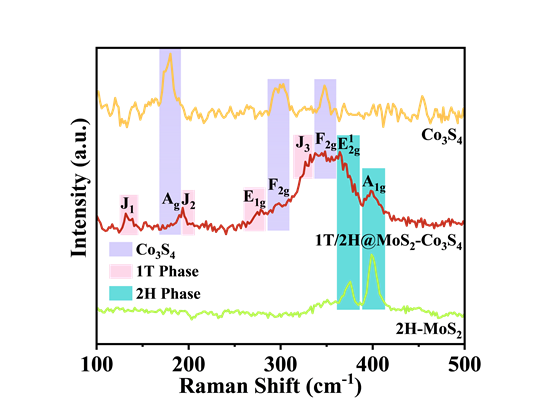
**

**Fig. S9** Raman spectra of different samples

**
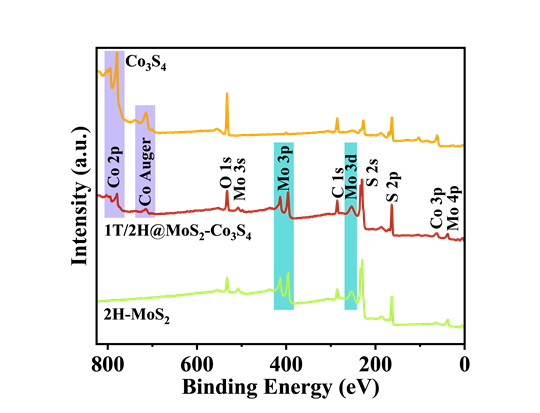
**

**Fig. S10** XPS survey spectra of different samples


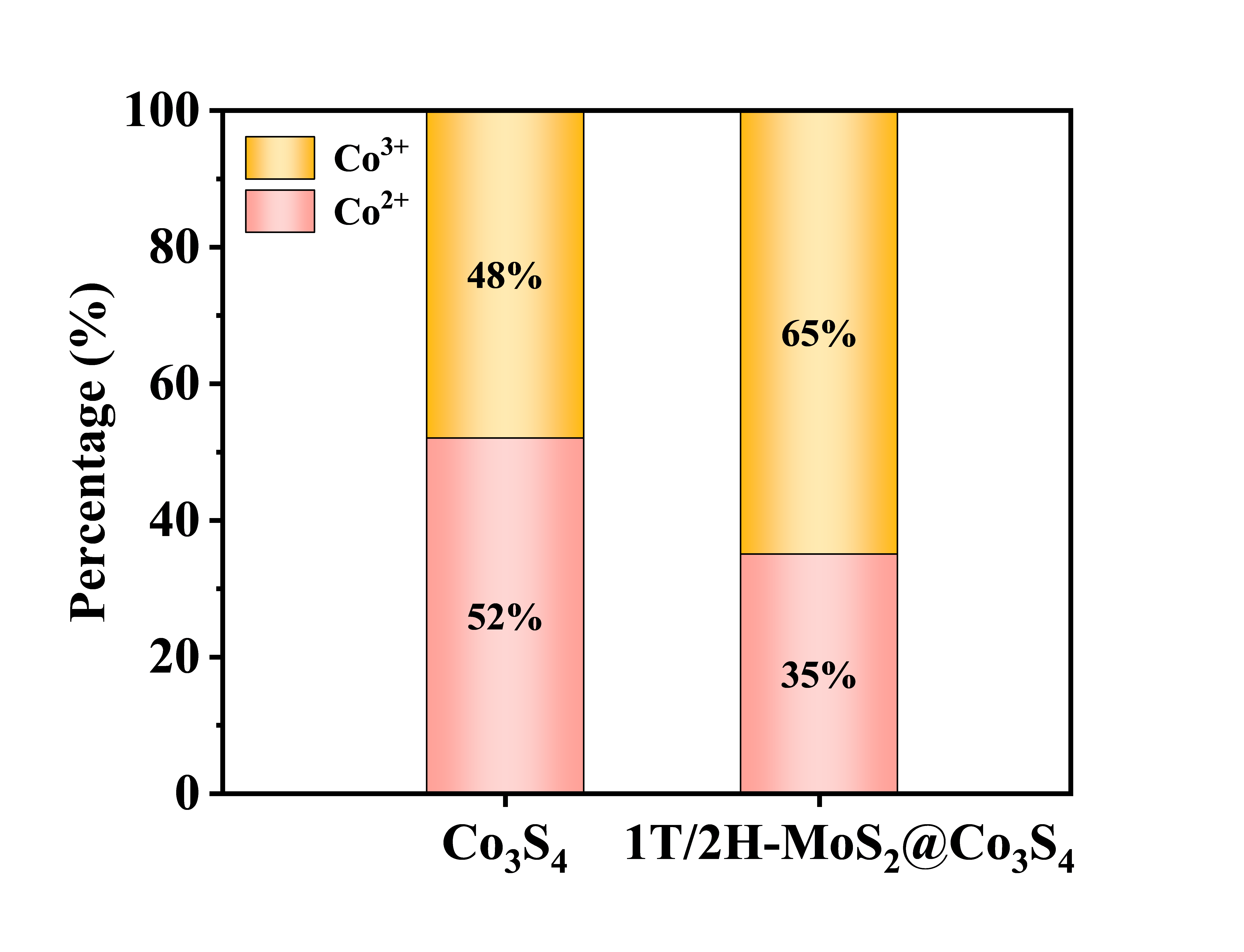


**Fig. S11** Percentages of Co^3+^ and Co^2+^ in 1T/2H-MCS and Co_3_S_4_

**
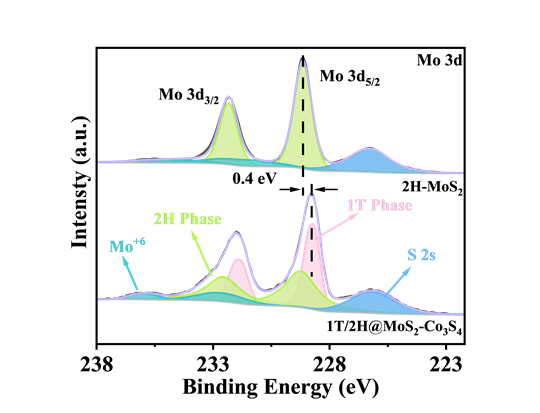
**

**Fig. S12** High-resolution Mo 3d XPS spectra of different samples

**
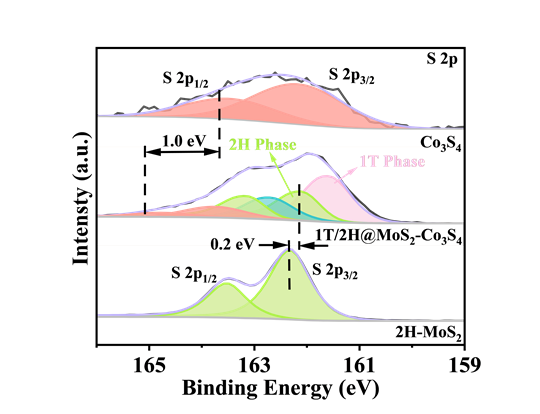
**

**Fig. S13** High-resolution S 2p XPS spectra of different samples

**
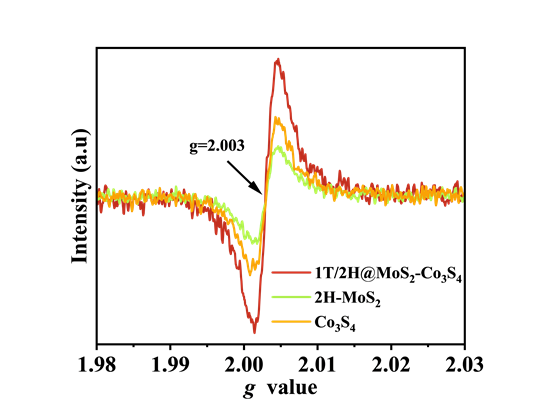
**

**Fig. S14** EPR spectra of different samples

**
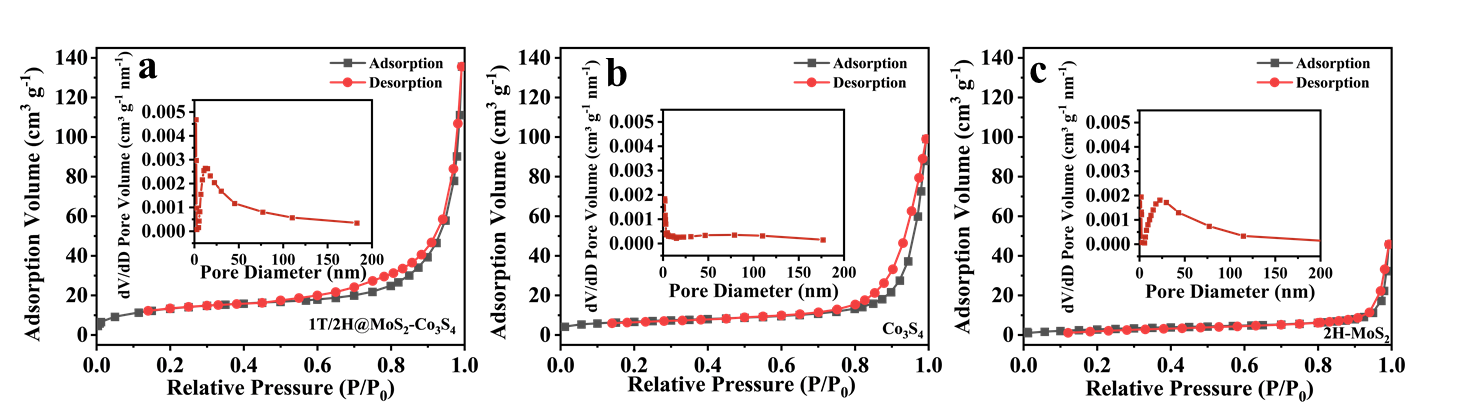
**

**Fig. S15** N_2_ adsorption-desorption isotherms with pore size distribution curves of **a** 1T/2H-MCS, **b** Co_3_S_4_, and **c** 2H-MoS_2_

**
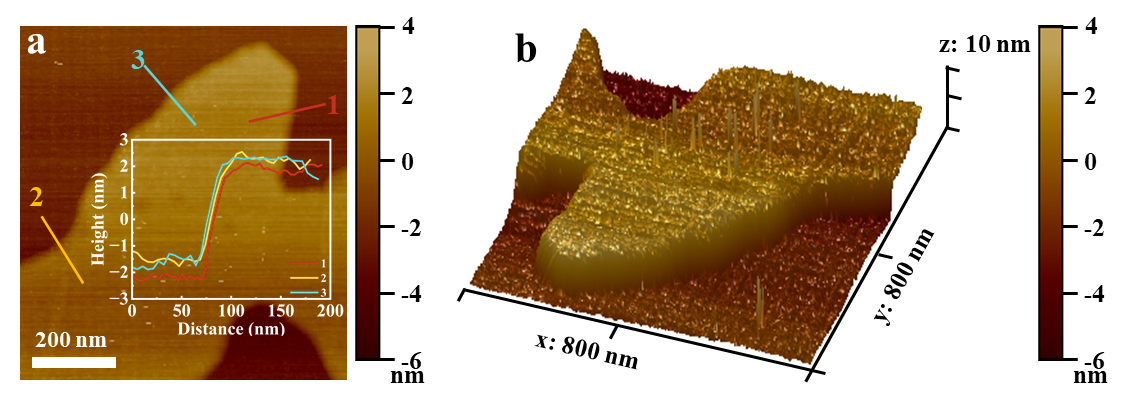
**

**Fig. S16 a** AFM image with **b** 3D photography of 1T/2H-MCS

**
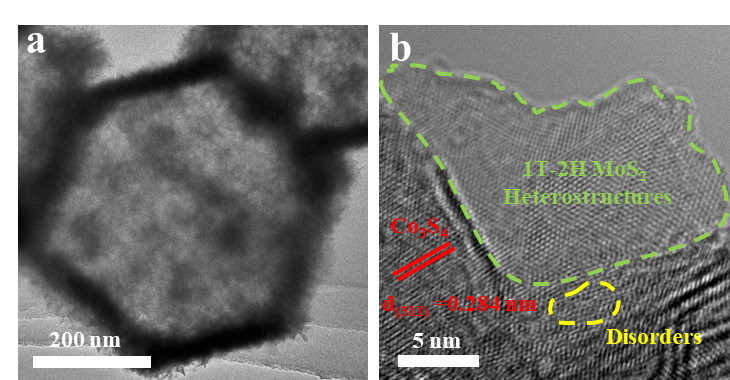
**

**Fig. S17** HRTEM images of 1T/2H-MCS

**
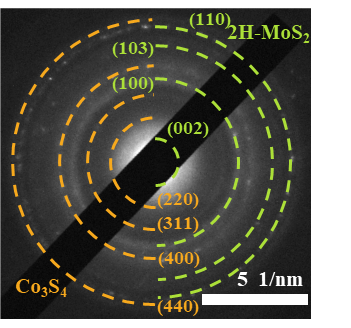
**

**Fig. S18** SAED pattern of 1T/2H-MCS


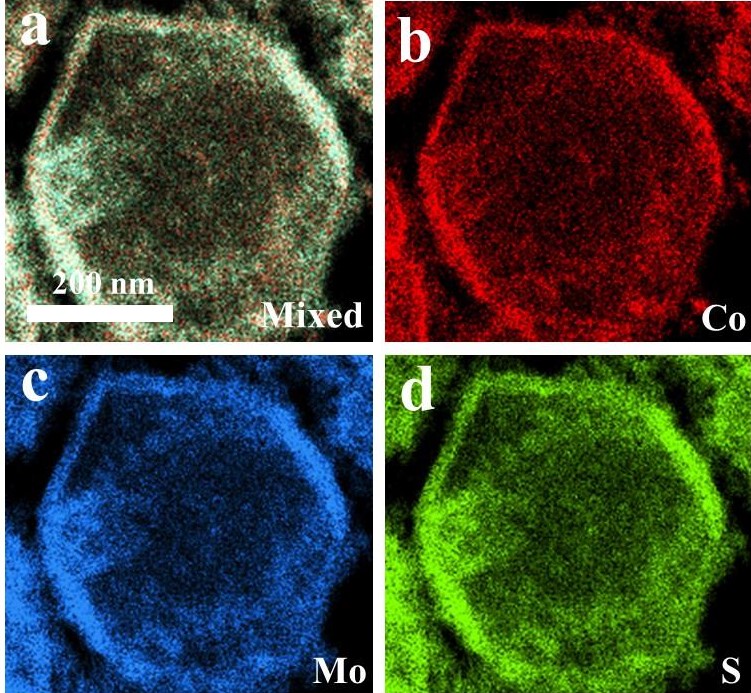


**Fig. S19** Element mapping images of 1T/2H-MCS

**
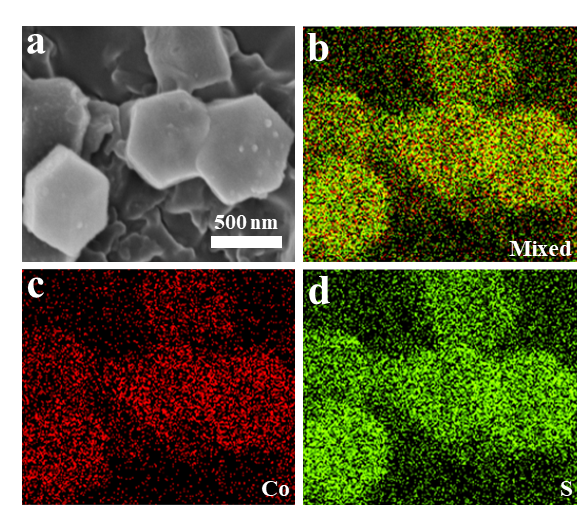
**

**Fig. S20 a** FESEM image with **b-d** corresponding element mapping images of Co_3_S_4_

**
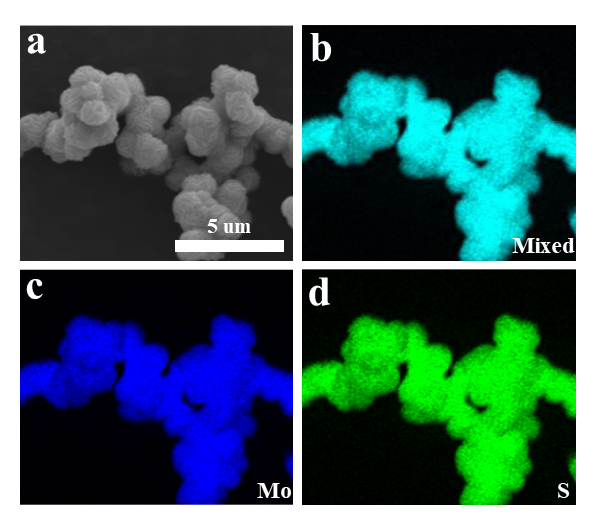
**

**Fig. S21 a** FESEM image with **b-d** corresponding element mapping images of 2H-MoS_2_

**
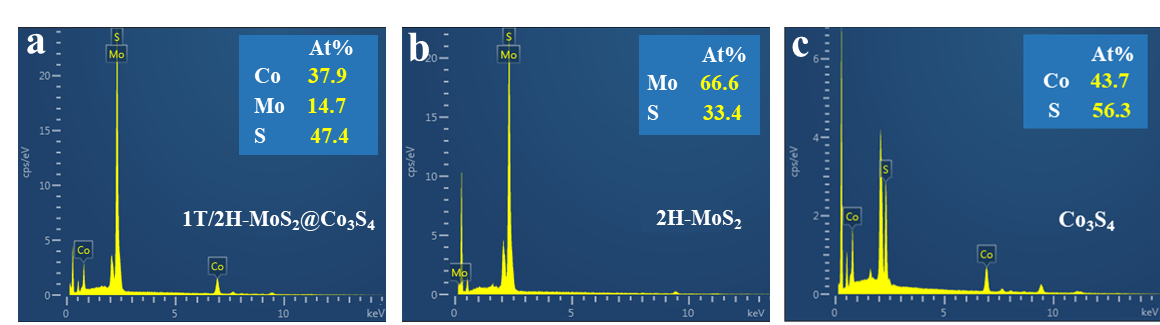
**

**Fig. S22** EDX results of **a** 1T/2H-MCS, **b** 2H-MoS_2_, and **c** Co_3_S_4_


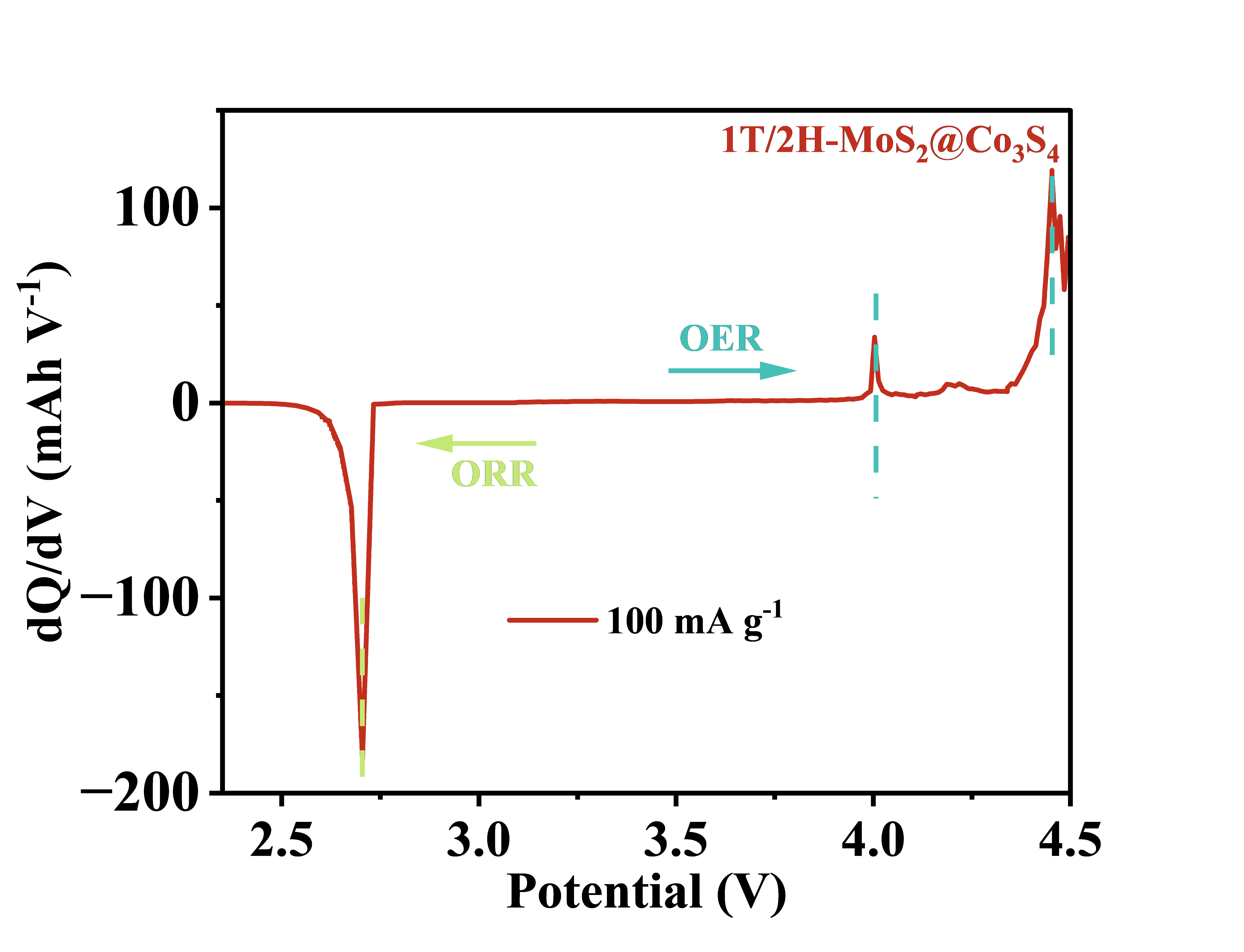


**Fig. S23** Differential profiles of 1T/2H-MCS cathode at 100 mA g^-1^


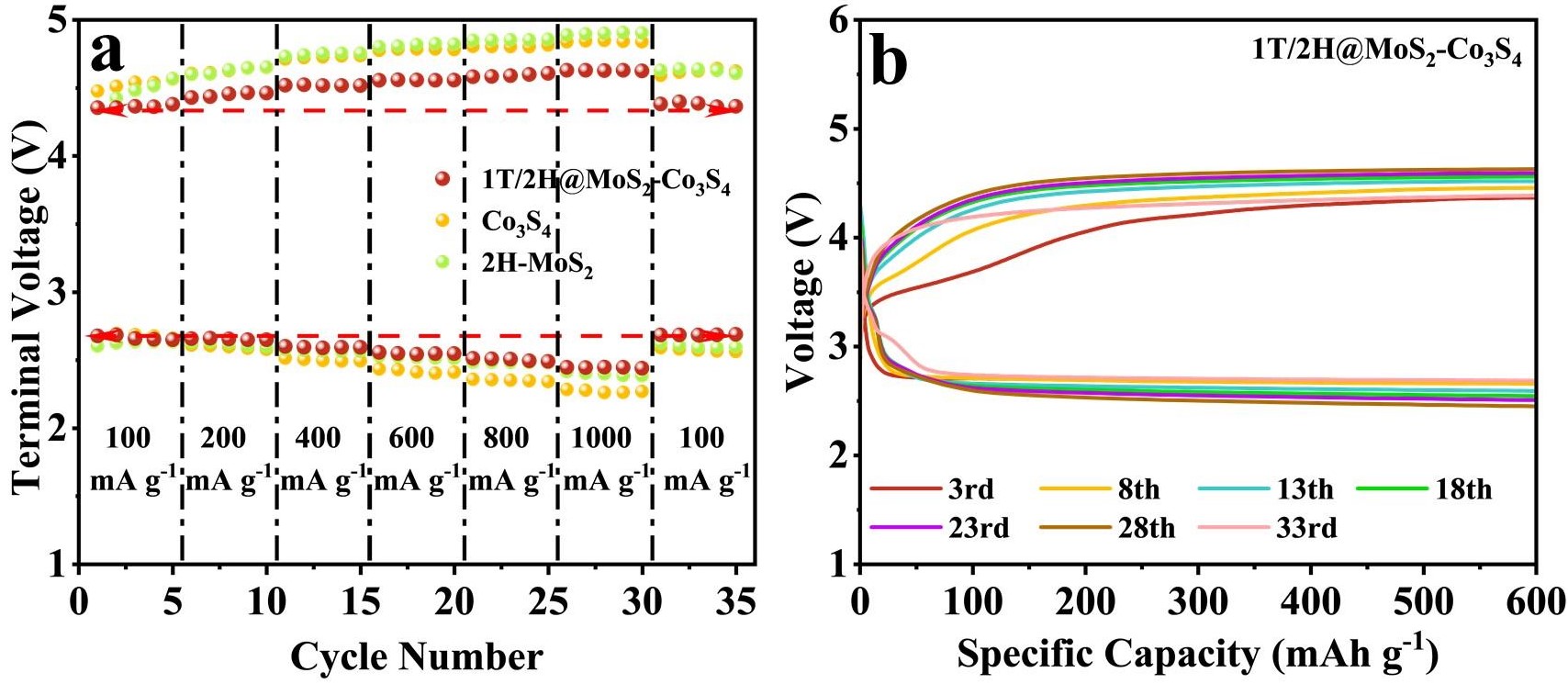


**Fig. S24 a** Rate performance of different cathodes and **b** corresponding typical discharge/charge curves of 1T/2H-MCS cathode


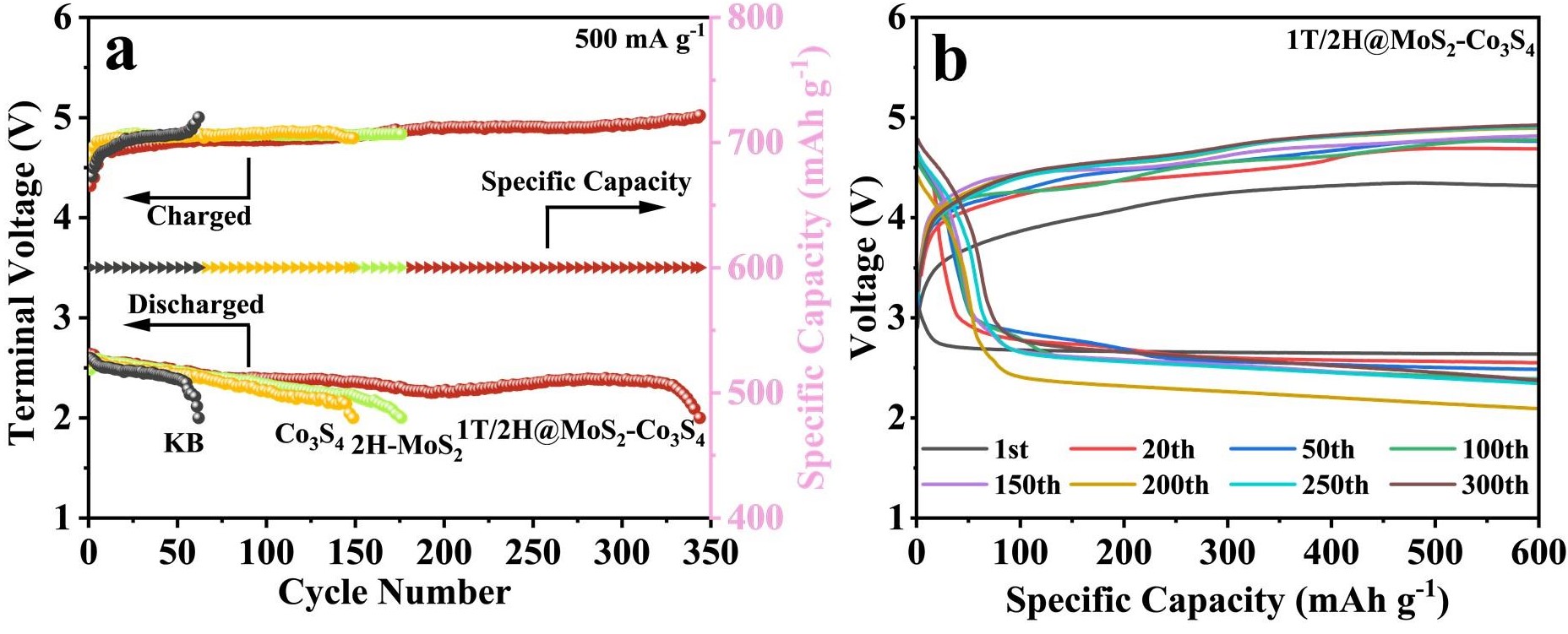


**Fig. S25 a** Cycling performance of different cathodes at 500 mA g^-1^ under a specific capacity limit of 600 mAh g^-1^ and **b** corresponding typical discharge/charge profiles of 1T/2H-MCS cathode

**
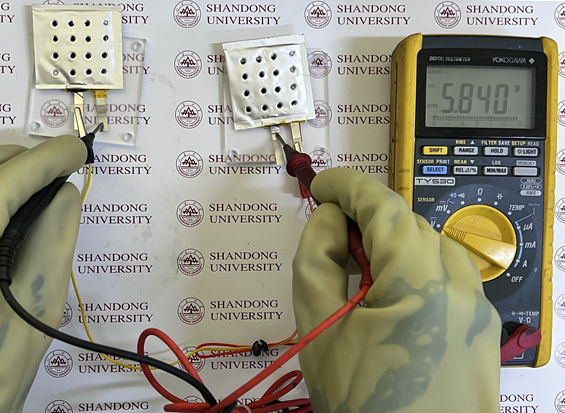
**

**Fig. S26** Open circuit voltage of pouch-type LOBs in series measured by a multimeter

**
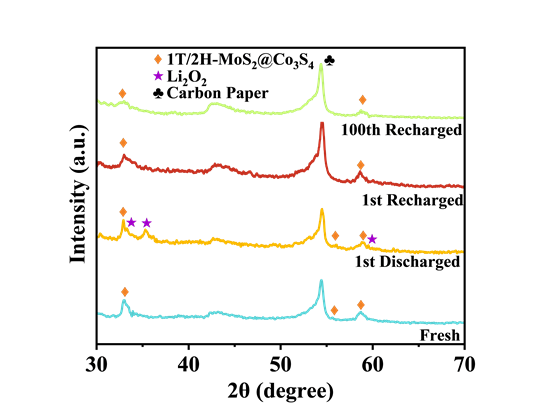
**

**Fig. S27** XRD patterns of 1T/2H-MCS cathodes at different stages


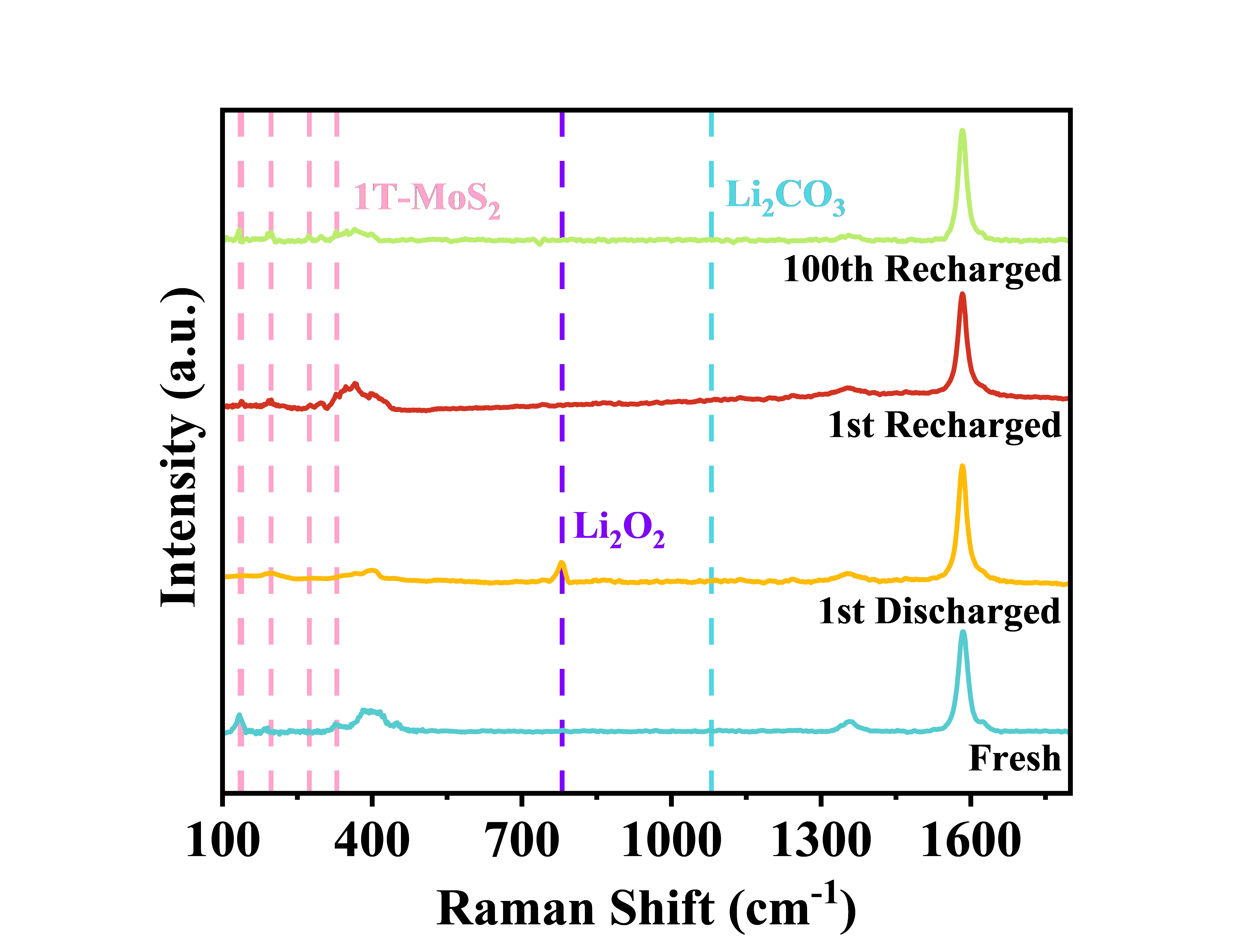


**Fig. S28** Raman spectra of 1T/2H-MCS cathodes at different stages

**
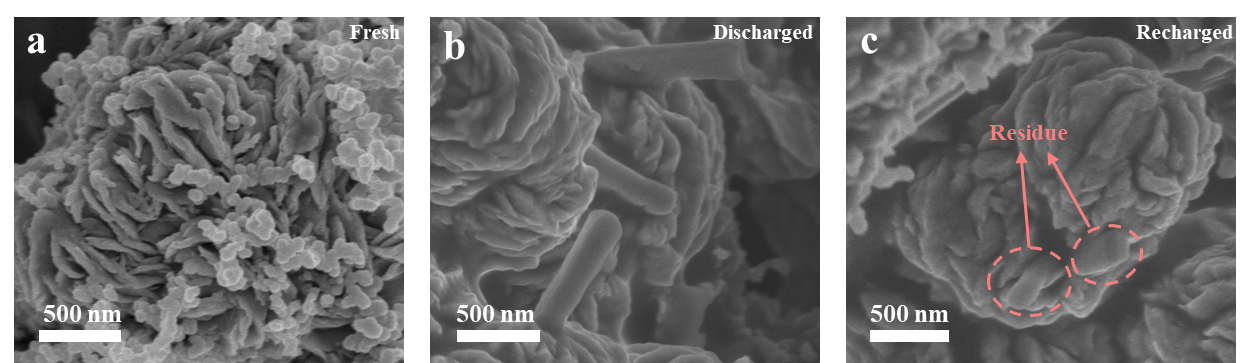
**

**Fig. S29** FESEM images of 2H-MoS_2_ cathodes **a** at fresh stage, **b** after 1st discharging, and **c** after 1st recharging


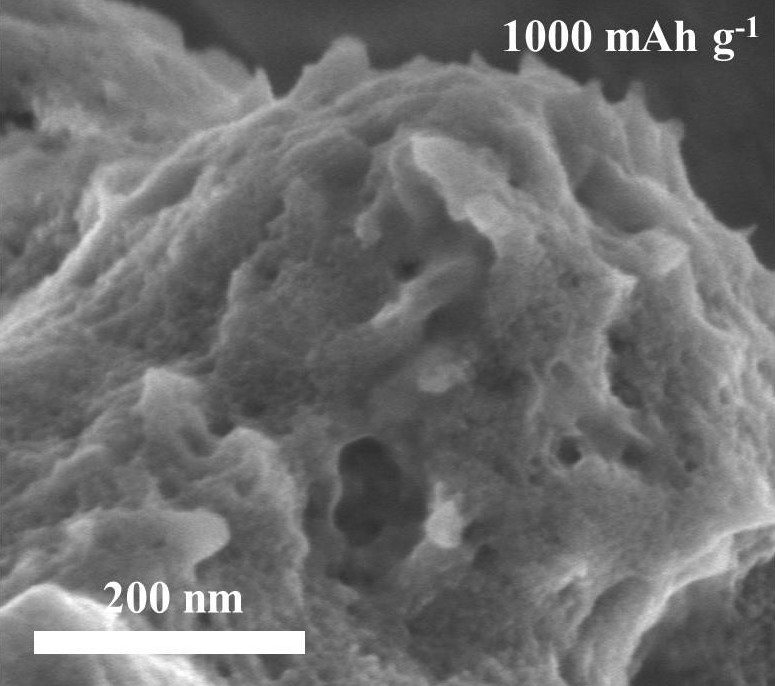


**Fig. S30** FESEM image of 1T/2H-MCS cathode after discharging to 1000 mAh g^-1^


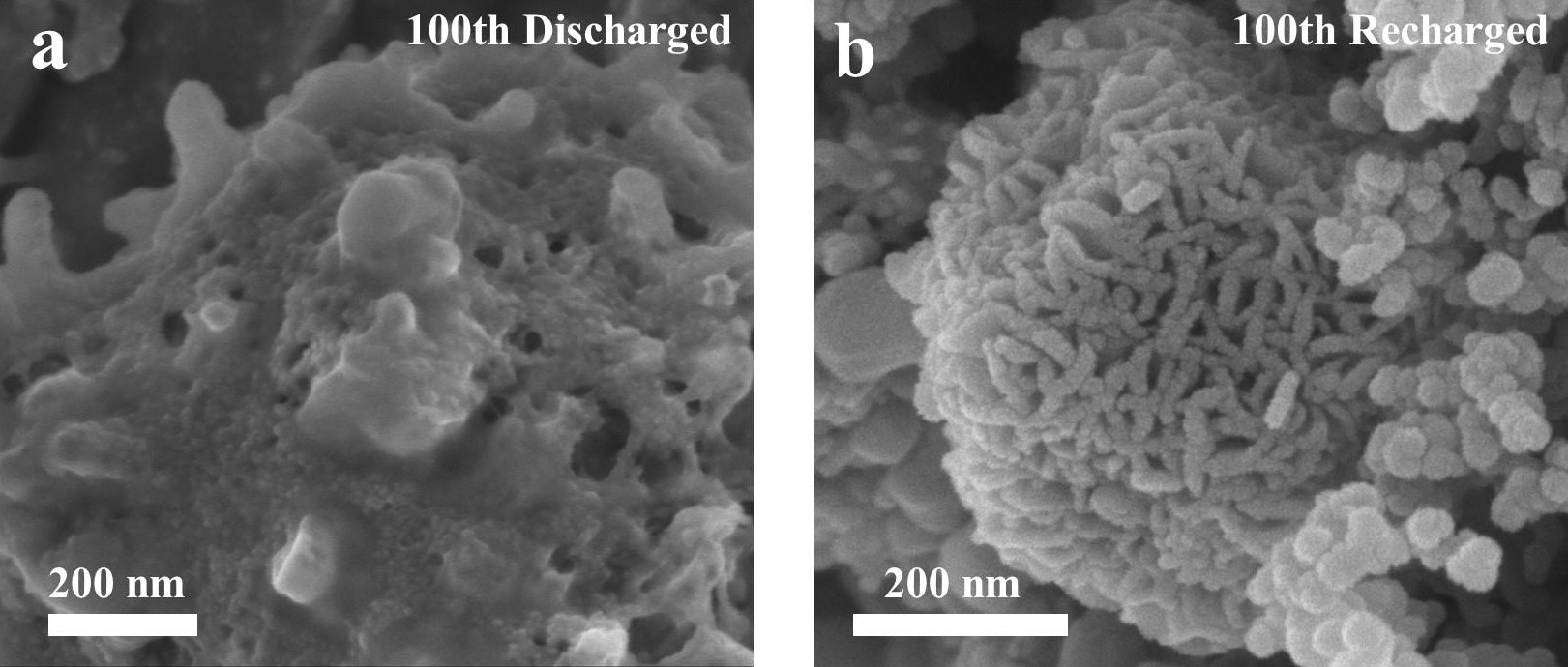


**Fig. S31** FESEM images of 1T/2H-MCS cathodes after **a** 100th discharging and **b** 100th recharging


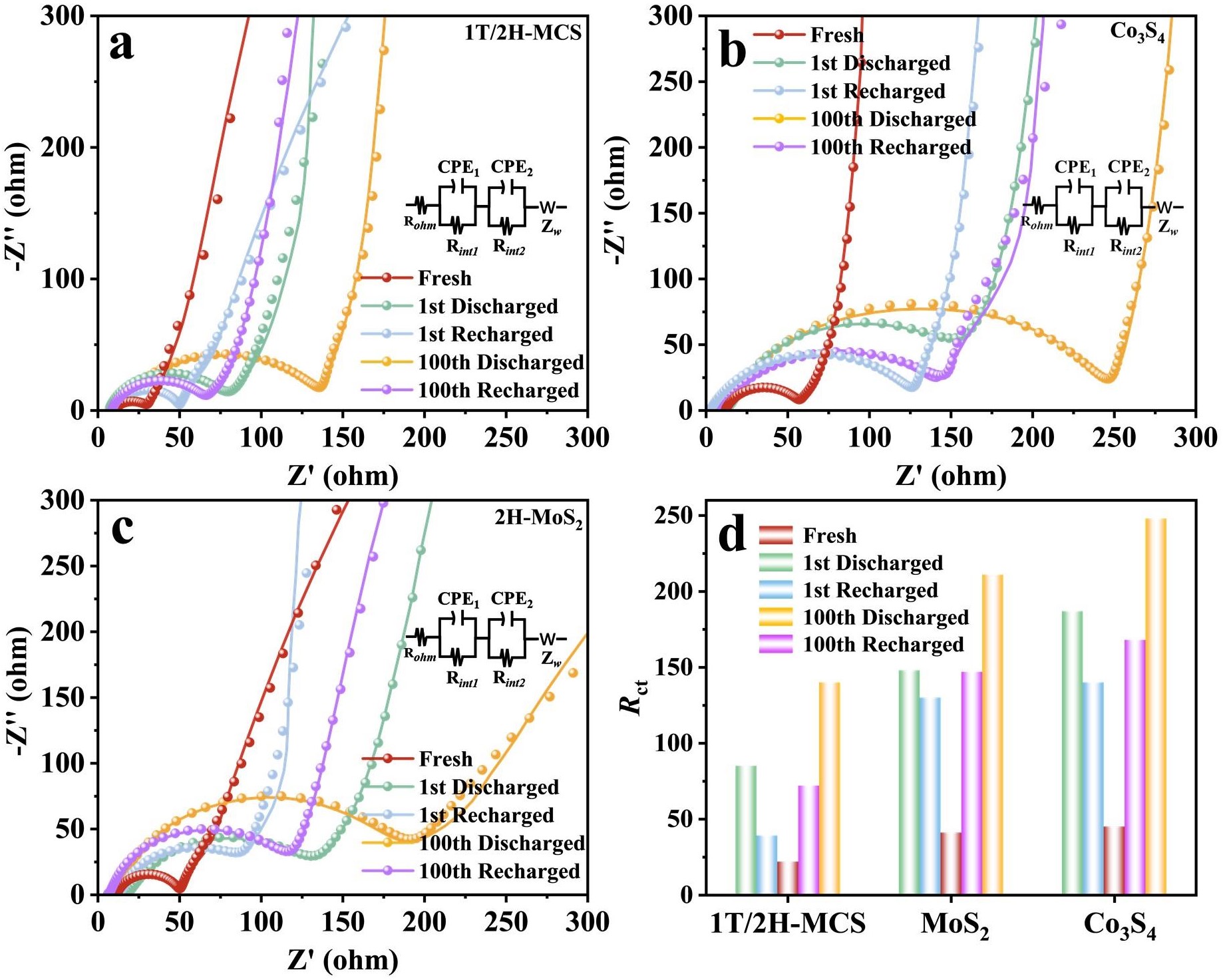


**Fig. S32** EIS plots of **a** 1T/2H-MCS, **b** Co_3_S_4_, and **c** 2H-MoS_2_ cathodes at different stages with equivalent circuits as insets and corresponding **d** charge transfer resistances of different cathodes

**
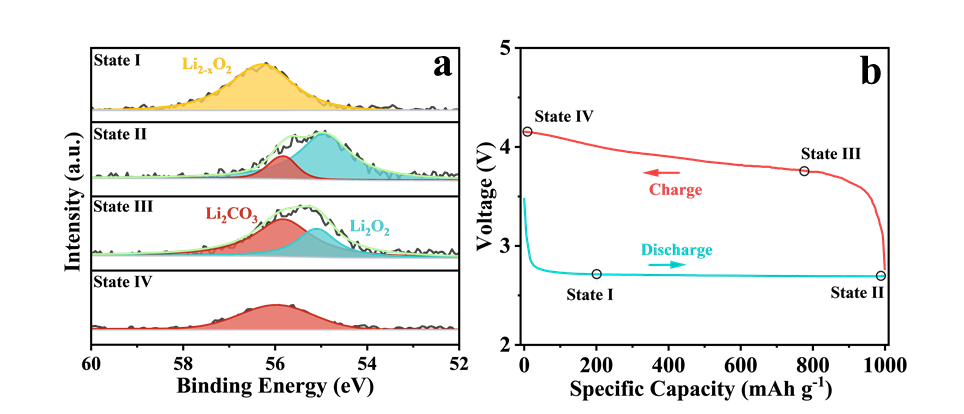
**

**Fig. S33 a** High-resolution Li 1s XPS spectra corresponding to States (I-IV) with **b** initial discharge-charge profiles of Co_3_S_4_ cathodes at 200 mA g^-1^ under a fixed specific capacity of 1000 mAh g^-1^

**
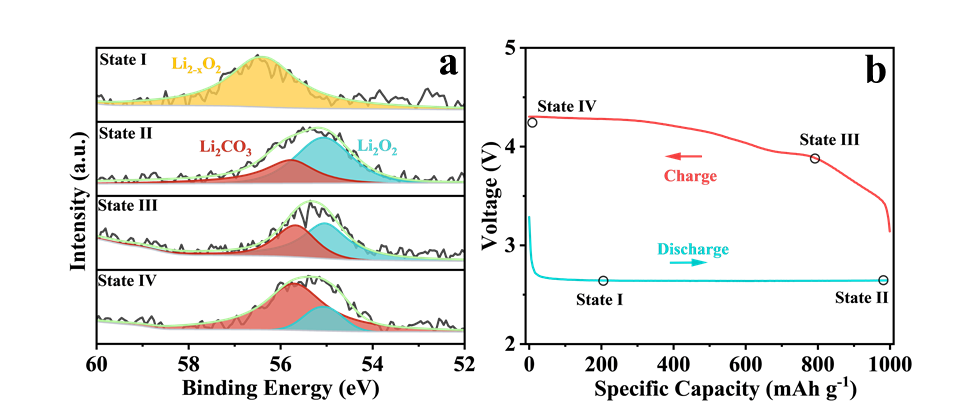
**

**Fig. S34** **a** High-resolution Li 1s XPS spectra corresponding to States I-IV with **b** initial discharge-charge profiles of 2H-MoS_2_ cathode at 200 mA g^-1^ under a fixed specific capacity of 1000 mAh g^-1^

**
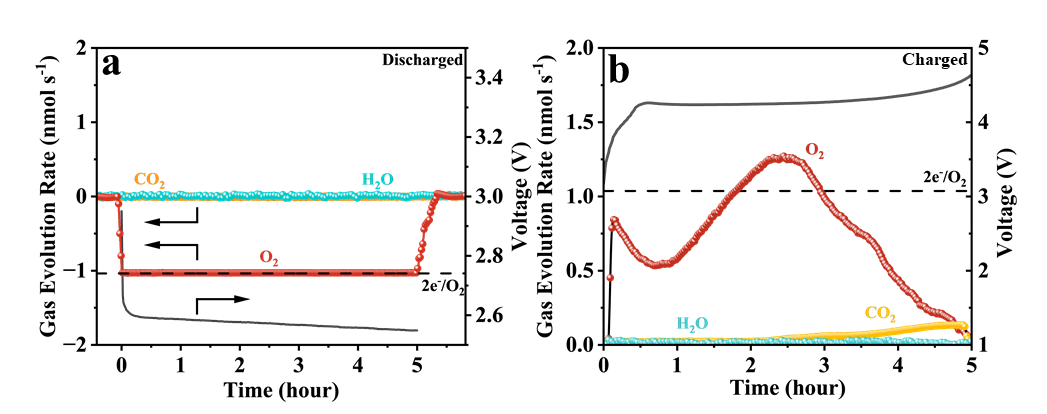
**

**Fig. S35** In-situ DEMS curves during **a** discharging and **b** charging at 200 mA g^-1^ of 2H-MoS_2_ cathodes

**
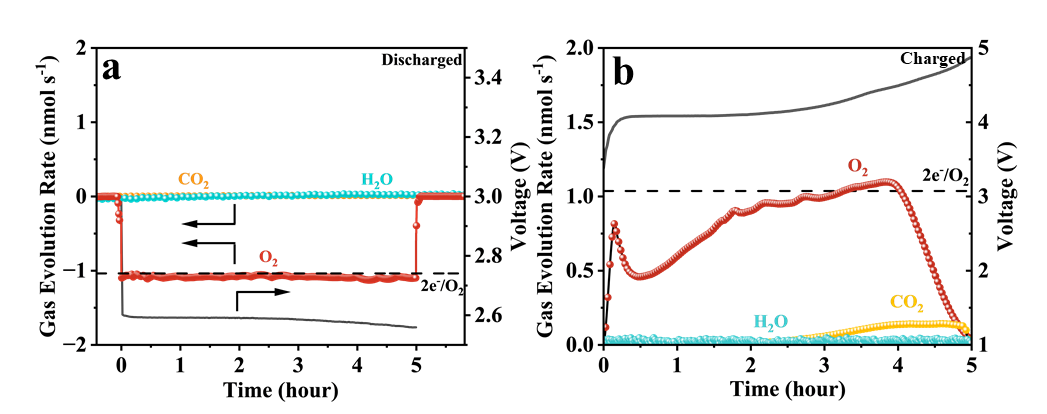
**

**Fig. S36** In-situ DEMS curves during **a** discharging and **b** charging at 200 mA g^-1^ of Co_3_S_4_ cathodes

**
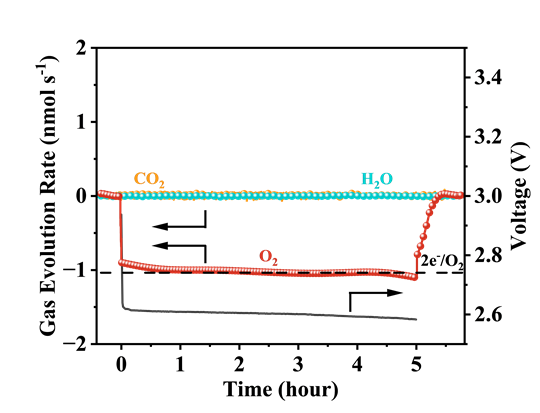
**

**Fig. S37** In-situ DEMS curves during discharging at 200 mA g^-1^ of 1T/2H-MCS cathode


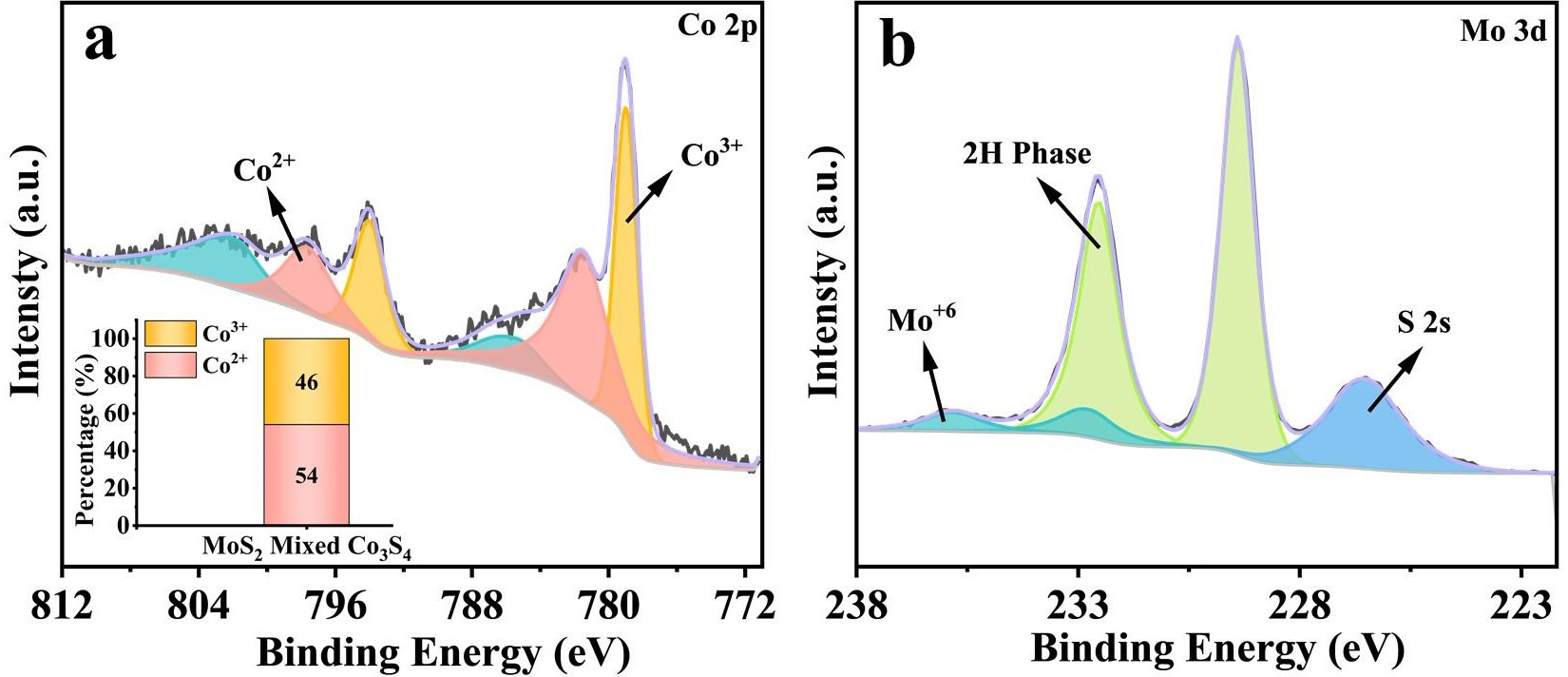


**Fig. S38** High-resolution **a** Co 2p and **b** Mo 3d XPS spectra of MoS_2_ Mixed Co_3_S_4_


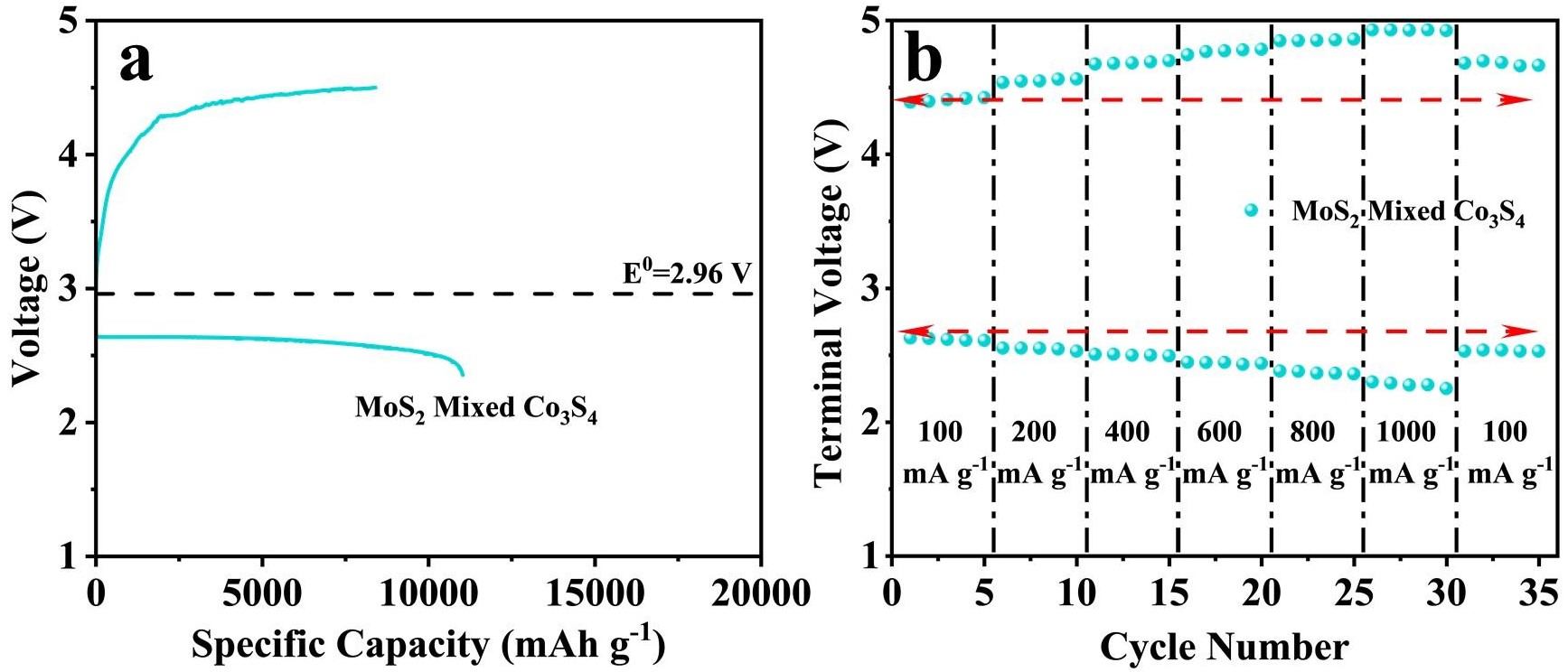


**Fig. S39 a** Initial discharge/charge curves at 100 mA g^-1^ and **b** rate performance of MoS_2_ Mixed Co_3_S_4_ cathodes


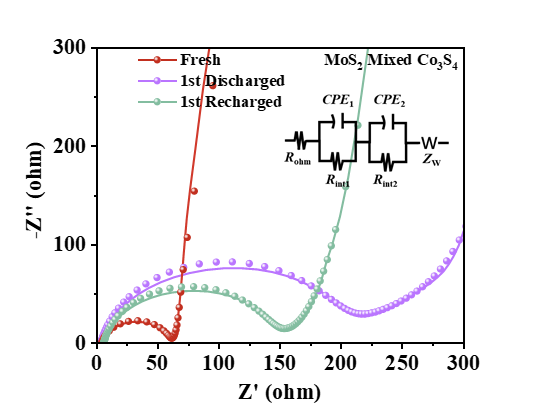


**Fig. S40** EIS plots of MoS_2_ mixed Co_3_S_4_ cathodes at different stages with the equivalent circuit as the inset

**
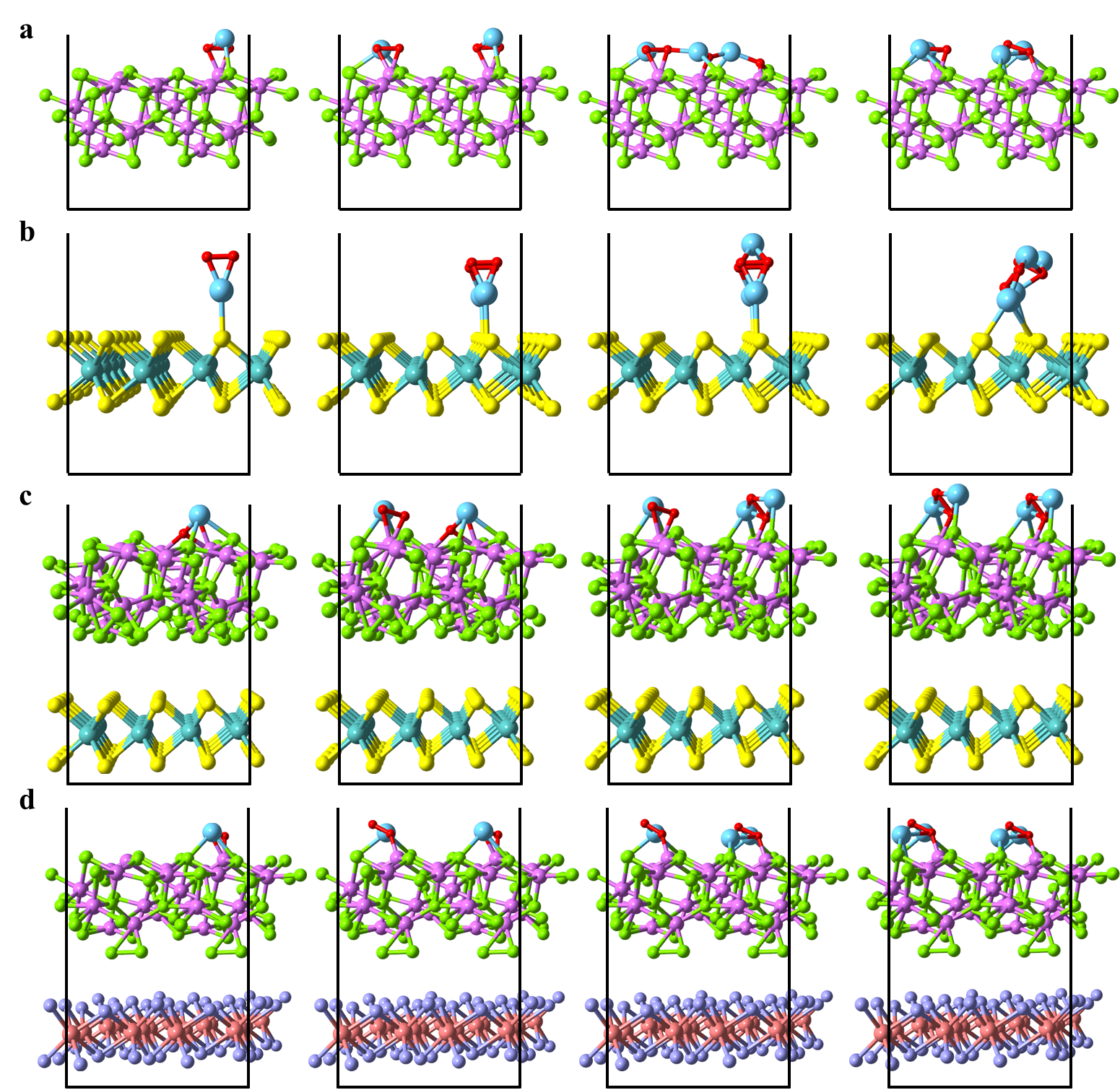
**

**Fig. S41** Optimized geometric structures of **a** Co_3_S_4_, **b** 2H-MoS_2_, **c** 2H-MoS_2_@Co_3_S_4_, and **d** 1T-MoS_2_@Co_3_S_4_ after adsorbing different oxygen species (O_2_, LiO_2_, Li_2_O_2_, and Li_4_O_4_)

**
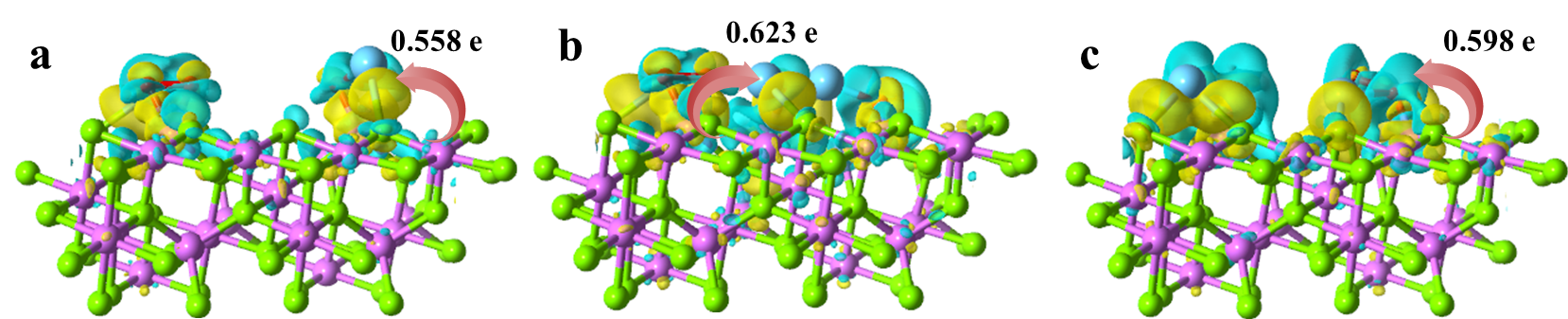
**

**Fig. S42** Charge density differences of Co_3_S_4_ after adsorbing **a** Li_2_O_4_^*^, **b** Li_3_O_4_^*^, and **c** Li_4_O_4_^*^

**
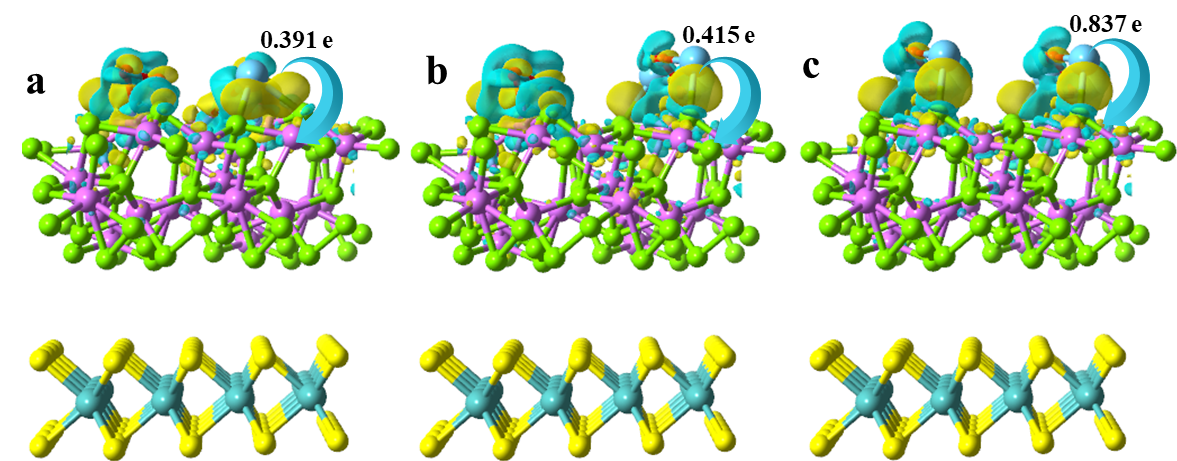
**

**Fig. S43** Charge density differences of 2H-MoS_2_@Co_3_S_4_ after adsorbing **a** Li_2_O_4_^*^, **b** Li_3_O_4_^*^, and **c** Li_4_O_4_^*^

**
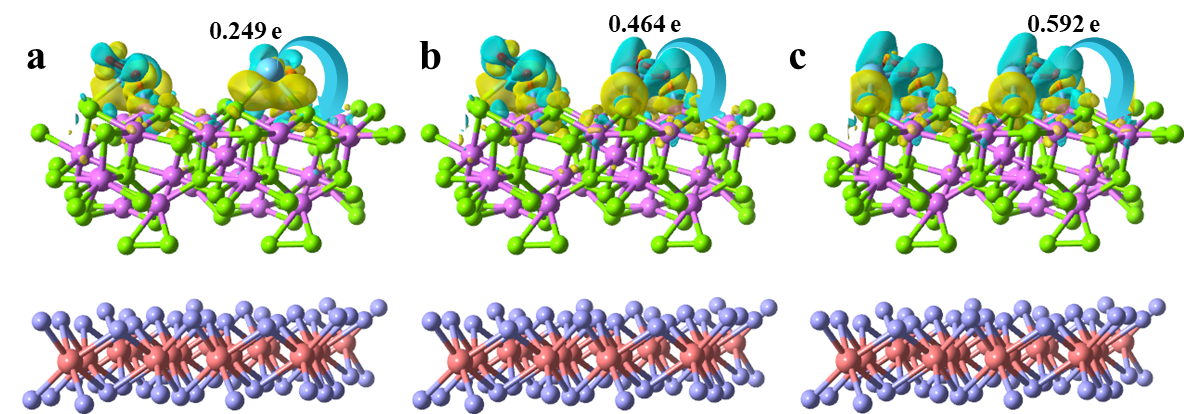
**

**Fig. S44** Charge density differences of 1T-MoS_2_@Co_3_S_4_ after adsorbing **a** Li_2_O_4_^*^, **b** Li_3_O_4_^*^, and **c** Li_4_O_4_^*^

**
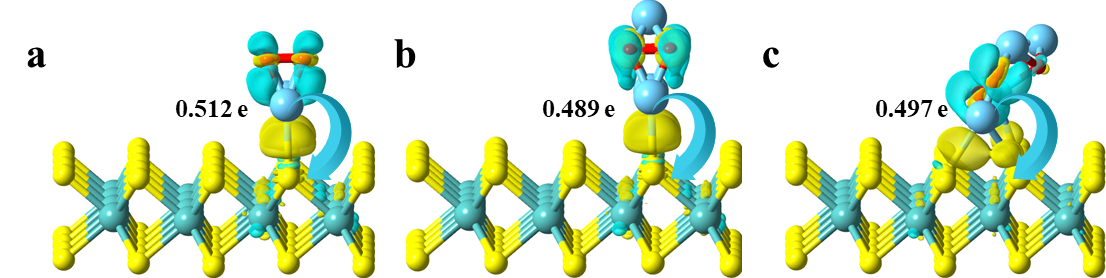
**

**Fig. S45** Charge density differences of 2H-MoS_2_ after adsorbing **a** Li_2_O_4_^*^, **b** Li_3_O_4_^*^, and **c** Li_4_O_4_^*^

**
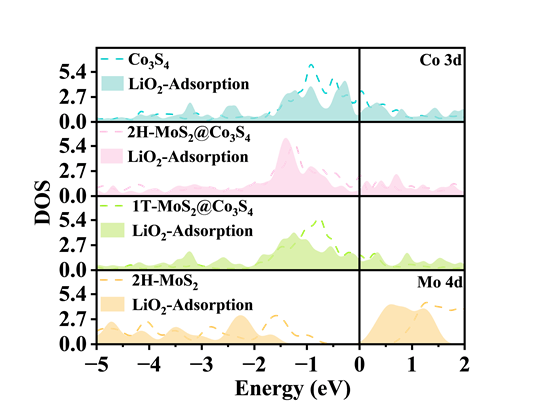
**

**Fig. S46** DOS of Co_3_S_4_, 2H-MoS_2_@Co_3_S_4_, 1T-MoS_2_@Co_3_S_4_, and 2H-MoS_2_ at fresh stage and after adsorbing LiO_2_^*^


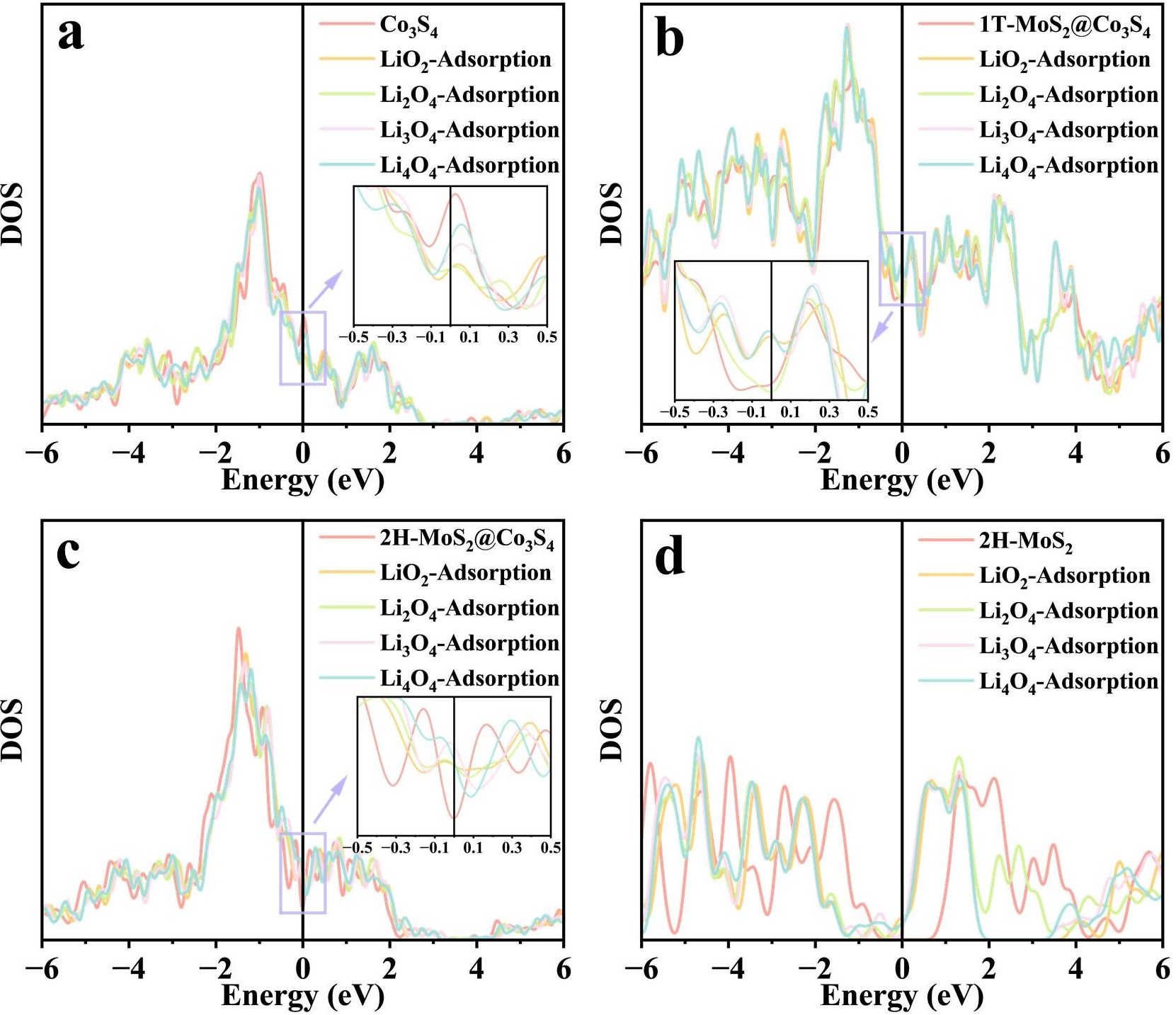


**Fig. S47** DOS data of **a** Co_3_S_4_, **b** 1T-MoS_2_@Co_3_S_4_, **c** 2H-MoS_2_@Co_3_S_4_, and **d** 2H-MoS_2_ adsorbed with different intermediates

**
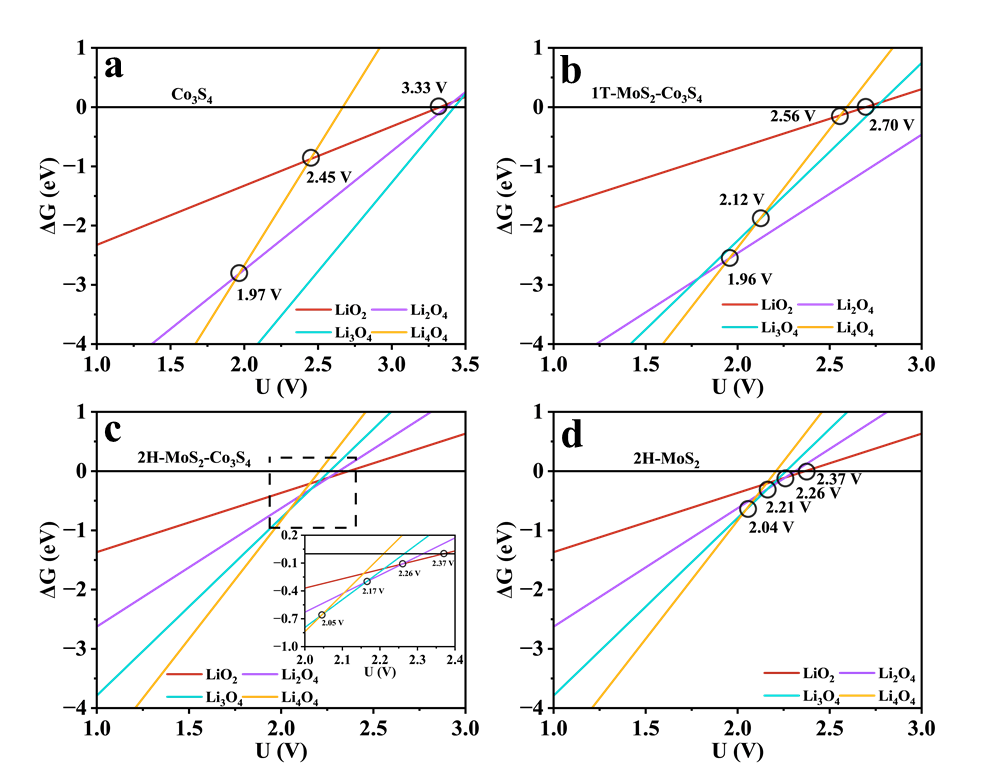
**

**Fig. S48** Phase diagrams of the cathode reactions on **a** Co_3_S_4_, **b** 1T-MoS_2_@Co_3_S_4_, **c** 2H-MoS_2_@Co_3_S_4_, and **d** 2H-MoS_2_ cathodes

**
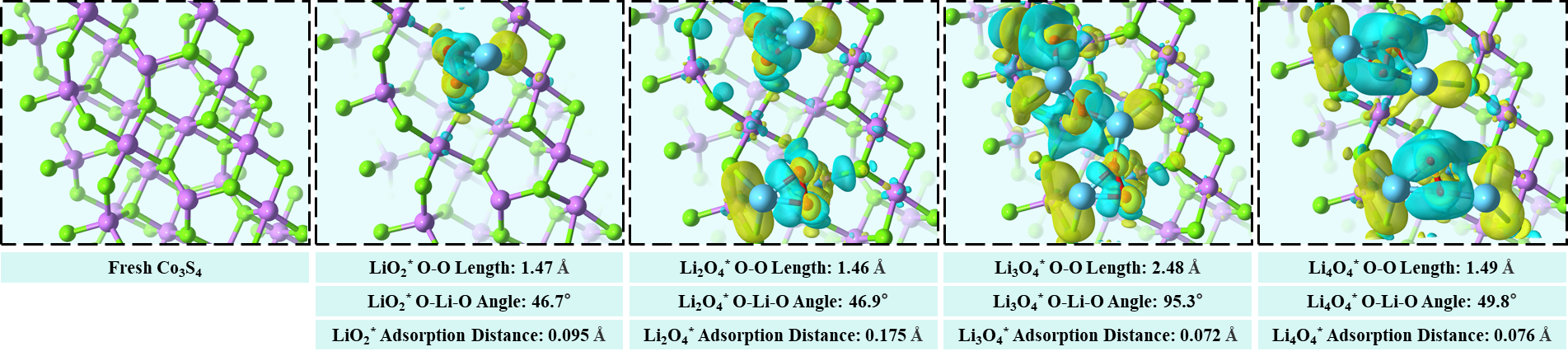
**

**Fig. S49** Optimized structure models (top sides) with the corresponding O-O bond lengths, O-Li-O angles, and adsorption distances for LiO_2_^*^/Li_2_O_4_^*^/Li_3_O_4_^*^/Li_4_O_4_^*^-adsorbed Co_3_S_4_ surfaces

**
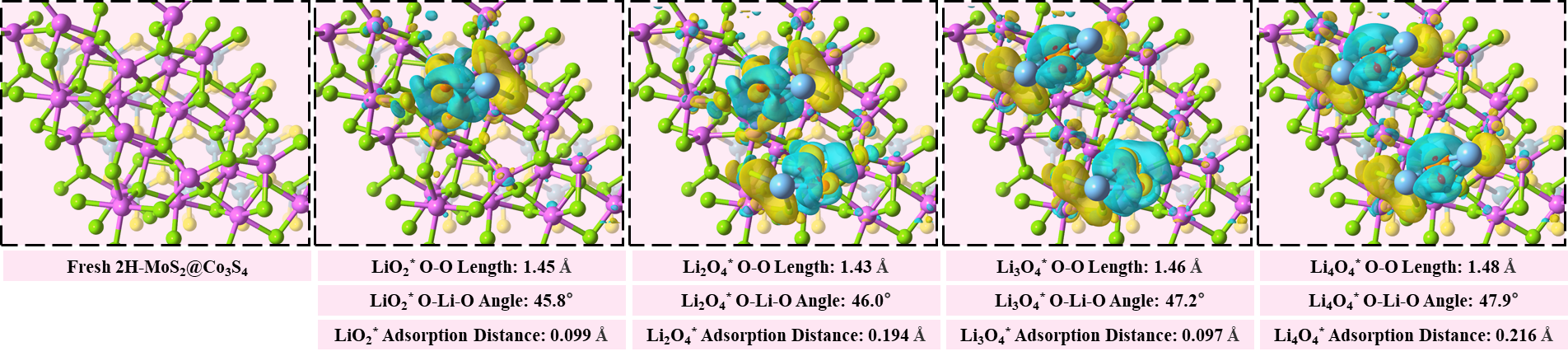
**

**Fig. S50** Optimized structure models (top sides) with the corresponding O-O bond lengths, O-Li-O angles, and adsorption distances for LiO_2_^*^/Li_2_O_4_^*^/Li_3_O_4_^*^/Li_4_O_4_^*^-adsorbed 2H-MoS_2_@Co_3_S_4_ surfaces

**
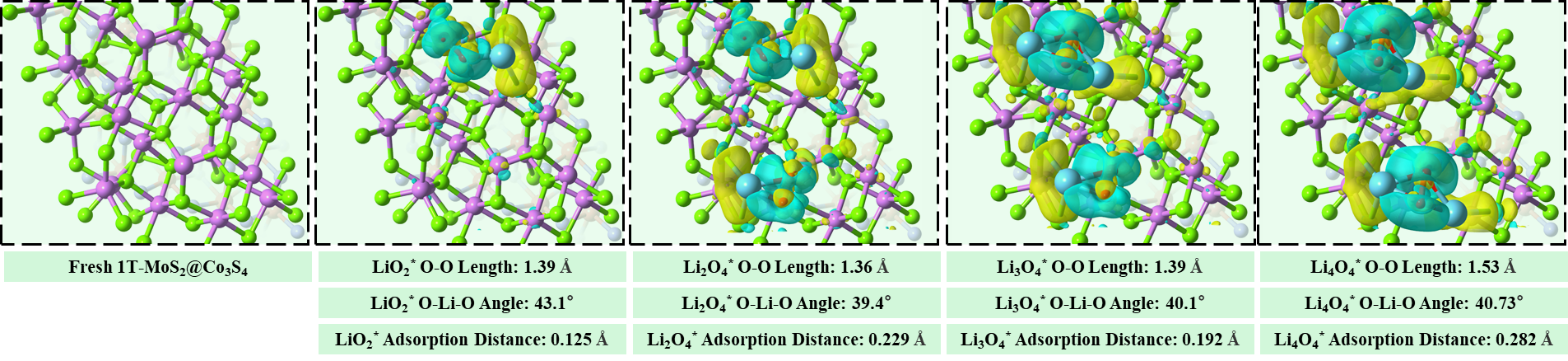
**

**Fig. S51** Optimized structure models (top sides) with the corresponding O-O bond lengths, O-Li-O angles, and adsorption distances for LiO_2_^*^/Li_2_O_4_^*^/Li_3_O_4_^*^/Li_4_O_4_^*^-adsorbed 1T-MoS_2_@Co_3_S_4_ surfaces

**
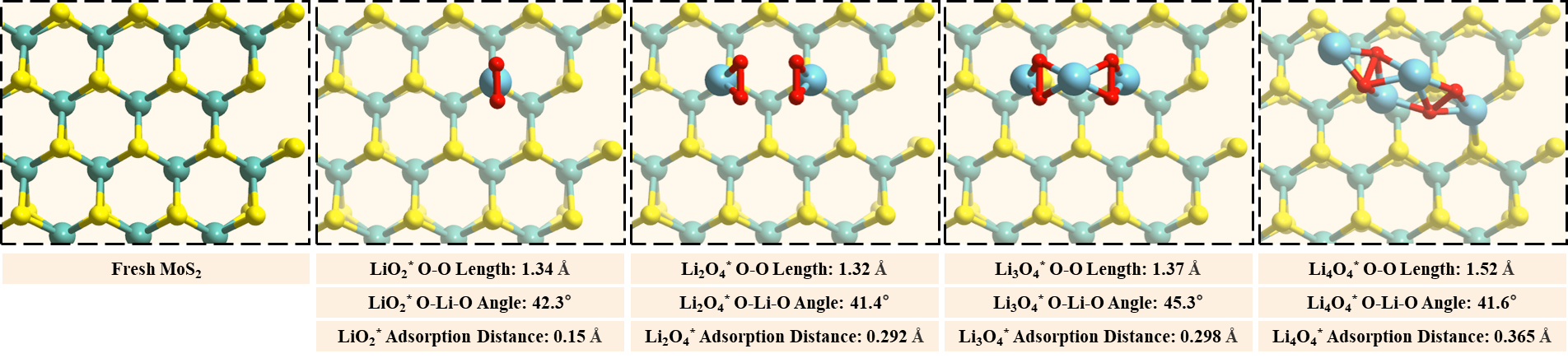
**

**Fig. S52** Optimized structure models (top sides) with the corresponding O-O bond lengths, O-Li-O angles, and adsorption distances for LiO_2_^*^/Li_2_O_4_^*^/Li_3_O_4_^*^/Li_4_O_4_^*^-adsorbed 2H-MoS_2_ surfaces

**Table S1** Parameters obtained from the fit of the Co K edge EXFAS spectra.

| **Catalysts** | **Path** | **N** | **R (Å)** | **ΔE^0^ (eV)** | **σ^2^ (Å^2^)** | **R-factor** |
| --- | --- | --- | --- | --- | --- | --- |
| 1T/2H-MCS | Co-S | 4 | 2.21(±0.02) | -4.9(±1.4) | 0.017(±0.005) | 0.008 |
|  | Co-Co | 8 | 4.00(±0.05) | -5.1(±2.2) | 0.033(±0.009) |  |
| Co_3_S_4_ | Co-S | 4 | 2.01(±0.01) | -0.5(±1.2) | 0.001(±0.002) | 0.005 |
|  | Co-Co | 8 | 3.69(±0.05) | -2.0(±0.7) | 0.065(±0.010) |  |
| Co Foil | Co-Co | 12 | 2.49(±0.01) | 6.8(±0.4) | 0.006(±0.001) | 0.001 |

**Table S2** Atomic ratios of Co_3_S_4_/MoS_2_ determined by ICP-OES

| **Sample** | **Atomic Percentage (%)** | | | **Atomic Ratio of Co_3_S_4_/MoS_2_** |
| --- | --- | --- | --- | --- |
|  | **Co** | **Mo** | **S** |  |
| 1T/2H-MCS | 32.88 | 10.45 | 56.67 | 51.2/48.8 |

**Table S3** Comparison of 1T/2H -MCS cathode with the representative and state-of-the-art cathodes containing Mo-, Co- and noble metal-based compounds

| **Materials** | **Current Density** (mA g^-1^)/  **Specific Capacity** (mAh g^-1^) | **Discharge/**  **Charge**  **Overpotential** (V) | **Coulombic**  **Eﬃciency** | **Current Density** (mA g^-1^)**-**  **Fixed Capacity** (mA g^-1^)/  **Cycle Hours** (h) | **Refs.** |
| --- | --- | --- | --- | --- | --- |
| 1T/2H-MCS | 100/18456 | 0.26/0.97 | 99.7% | 200 -1000/2420 | This  Work |
| Co_3_S_4_ | 100/5917 | 0.26/1.10 | 95.2% | 100-500/250 | [S1] |
| Ni-RuO_2_ | 200/19698 | 0.28/1.19 | - | 500-500/1580 | [S2] |
| Co-S@NC | 1000/34587 | 0.21/0.64 | 110% | 500-1000/1240 | [S3] |
| Fe_SA_-RuO_2_/HPCS | 200/23628 | 0.26/0.89 | - | 200-1000/2300 | [S4] |
| Ru/O-MoS_2_ | 100/18700 | 0.17/0.84 | 96.2% | 200-1000/1200 | [S5] |
| Pt/RuO_2_/G | 200/4.3 mAh cm^-2^ | 0.25/1.22 | 104.6% | 200-1000/2200 | [S6] |
| PtAu | 100/5049 | 0.24/1.23 | 99.2% | 500-1000/880 | [S7] |
| MnS-MoS_2_ | 100/11696 | 0.22/1.20 | 95.2% | 100-800/1800 | [S8] |
| RuFe@NC | 150/11102 | 0.33/1.27 | 99.7% | 150-1000/1240 | [S9] |
| Pd_1_Co_2_P*_x_* | 200/6825 | 0.27/1.15 | - | 200-500/1200 | [S10] |
| P-NiCo_2_S_4_ | 100/17392 | 0.39/1.49 | 86.8% | 500-600/1296 | [S11] |
| P-CuMo_2_S_4_ | 100/13700 | 0.27/1.23 | 98.5% | 200-600/1020 | [S12] |
| CoPP-O-MXene | 100/11035 | 0.32/1.23 | 95.5% | 300-500/1433 | [S13] |
| Ti_0.87_O_2_/MXene | 100/13500 | 0.31/1.27 | 98.4% | 500-600/982 | [S14] |
| CNTs@Co_4_N@C | 200/10000 | 0.22/0.91 | - | 200-500/1250 | [S15] |
| MoO_2_/Mo_3_P | 50/4720 | 0.26/0.92 | - | 500-500/1000 | [S16] |
| RuPd/Fe-CNCs | 200/31211 | 0.24/0.60 | - | 200-1000/3000 | [S17] |

**Supplementary References**

1. P. Sennu, M. Christy, V. Aravindan, Y.-G. Lee, K.S. Nahm et al., Two-dimensional mesoporous cobalt sulfide nanosheets as a superior anode for a Li-ion battery and a bifunctional electrocatalyst for the Li–O2 system. Chem. Mater. **27**(16), 5726–5735 (2015). <https://doi.org/10.1021/acs.chemmater.5b02364>
2. C. Sun, X. Cui, F. Xiao, D. Cui, Q. Wang et al., Modulating the d-band center of RuO_2_ *via* Ni incorporation for efficient and durable Li–O2 batteries. Small **20**(32), 2400010 (2024). <https://doi.org/10.1002/smll.202400010>
3. N. Meng, Y. Feng, Z. Zhao, F. Lian, Boosting the ORR/OER activity of cobalt-based nano-catalysts by Co_3_ d orbital regulation. Small **20**(35), 2400855 (2024). <https://doi.org/10.1002/smll.202400855>
4. Z. Lian, Y. Lu, S. Zhao, Z. Li, Q. Liu, Engineering the electronic interaction between atomically dispersed Fe and RuO_2_ attaining high catalytic activity and durability catalyst for Li-O2 battery. Adv. Sci. **10**(9), 2205975 (2023). <https://doi.org/10.1002/advs.202205975>
5. X. Cao, M. Cui, K. Fang, L. Yan, H. Gong et al., Ruthenium atoms anchored on oxygen-modified molybdenum disulfide with strong interfacial coupling as efficient and stable catalysts for lithium–oxygen batteries. J. Colloid Interface Sci. **679**, 234–242 (2025). <https://doi.org/10.1016/j.jcis.2024.09.226>
6. Y. Li, Y. Li, Y. Ding, J. Ma, P. Das et al., Spatially confined sub-nanometer Pt in RuO_2_ nanosheet as robust bifunctional oxygen electrocatalyst for stabilizing Li-O2 batteries. Chem Catal. **3**(9), 100658 (2023). <https://doi.org/10.1016/j.checat.2023.100658>
7. Y. Zhou, Q. Gu, K. Yin, Y. Li, L. Tao et al., Engineering eg orbital occupancy of Pt with Au alloying enables reversible Li−O2 batteries. Angew. Chem. Int. Ed. **61**(26), e202201416 (2022). <https://doi.org/10.1002/anie.202201416>
8. G. Zhang, H. Yu, X. Li, X. Zhang, C. Hou et al., Construction of MnS/MoS_2_ heterostructure on two-dimensional MoS_2_ surface to regulate the reaction pathways for high-performance Li-O2 batteries. J. Energy Chem. **93**, 443–452 (2024). <https://doi.org/10.1016/j.jechem.2024.01.076>
9. J. Hong, S. Hyun, M. Tsipoaka, J.S. Samdani, S. Shanmugam, RuFe alloy nanoparticle-supported mesoporous carbon: efficient bifunctional catalyst for Li-O2 and Zn–air batteries. ACS Catal. **12**(3), 1718–1731 (2022). <https://doi.org/10.1021/acscatal.1c04527>
10. Z. Xu, Y. Zhang, H. Yu, Z. Zhuang, X. Wang et al., Interface engineering of branched PdCoP(x) nanostructures for high-performance lithium-oxygen batteries. Angew. Chem. Int. Ed. **64**(30), e202504924 (2025). <https://doi.org/10.1002/anie.202504924>
11. S. Shoukat, Y. Dou, M. Gao, D. Wan, M. Shoaib et al., Tuning electronic properties of NiCo2S4 nanofibers through phosphorous doping for optimized oxygen reduction and evolution reactions in Li-O2 batteries. Chem. Eng. J. **513**, 162908 (2025). <https://doi.org/10.1016/j.cej.2025.162908>
12. Y. Zhang, Q. Xia, J. Wang, L. Zhao, Z. Zhang et al., Electrocatalysis-dependent dynamic surface reconstruction of redox couples for bifunctional electrocatalysts. Appl. Catal. B Environ. Energy **376**, 125468 (2025). <https://doi.org/10.1016/j.apcatb.2025.125468>
13. P. Liu, C. Wang, C. Zeng, S. Wang, X. Yu et al., Single-atom sites with axial ligand-induced d orbital rearrangement as efficient electrocatalysts for lithium–oxygen batteries. SusMat **5**(3), e70007 (2025). <https://doi.org/10.1002/sus2.70007>
14. D. Zhang, G. Zhang, R. Liu, R. Yang, X. Li et al., Mutually activated 2D Ti_0.87_O_2_/MXene monolayers through electronic compensation effect as highly efficient cathode catalysts of Li–O2 batteries. Adv. Funct. Mater. **35**(5), 2414679 (2025). <https://doi.org/10.1002/adfm.202414679>
15. J. Xia, S. Yin, K. Cui, T. Yang, Y. Yan et al., Self-catalyzed growth of co(4)N and N-doped carbon nanotubes toward bifunctional cathode for highly safe and flexible Li-air batteries. ACS Nano **18**(16), 10902–10911 (2024). <https://doi.org/10.1021/acsnano.4c01271>
16. T. Yang, Y. Xia, T. Mao, Q. Ding, Z. Wang et al., Phosphorus vacancies and heterojunction interface as effective lithium-peroxide promoter for long-cycle life lithium–oxygen batteries. Adv. Funct. Mater. **32**(49), 2209876 (2022). <https://doi.org/10.1002/adfm.202209876>
17. L. Huang, L. Zhang, M. Liu, Q. Zhang, Z. Chen et al., Nitrogen and atomic Fe Co-doped hollow carbon nanocages supporting RuPd nanoclusters as extraordinary high-performance nanoreactor-like cathode for lithium–oxygen batteries. Energy Storage Mater. **61**, 102874 (2023). <https://doi.org/10.1016/j.ensm.2023.102874>
